# Varenicline and Nicotine Replacement Therapy for Smokers Admitted to Hospitals: A Randomized Clinical Trial

**DOI:** 10.1001/jamanetworkopen.2024.18120

**Published:** 2024-06-27

**Authors:** Gregory R. Weeks, Rukshar K. Gobarani, Michael J. Abramson, Billie Bonevski, Ashley Webb, Dennis Thomas, Eldho Paul, Muhammad R. Sarwar, Brian J. Smith, Sharmilla Perinpanathan, Sue Kirsa, Jacqueline Parkinson, Darshana Meanger, Lisa Coward, Olivia Rofe, Paula Lee, Denise van den Bosch, Johnson George

**Affiliations:** 1 Centre for Medicine Use and Safety, Faculty of Pharmacy and Pharmaceutical Sciences, Monash University, Parkville, Victoria, Australia; 2 Pharmacy Department, Barwon Health, Geelong, Victoria, Australia; 3 School of Public Health and Preventive Medicine, Monash University, Melbourne, Victoria, Australia; 4 Flinders Health and Medical Research Institute, College of Medicine and Public Health, Flinders University, Bedford Park, South Australia; 5 Department of Anaesthesia and Pain Management, Peninsula Health, Frankston, Victoria, Australia; 6 Centre of Excellence in Treatable Traits, College of Health, Medicine and Wellbeing, Hunter Medical Research Institute, University of Newcastle, Newcastle, New South Wales, Australia; 7 General and Respiratory Medicine, Bendigo Hospital, Spring Gully, Victoria, Australia; 8 Monash Health, Clayton, Victoria, Australia; 9 Pharmacy Department, Eastern Health, Box Hill, Victoria, Australia

## Abstract

**Importance:**

Varenicline is the most effective sole pharmacotherapy for smoking cessation. If used in combination with nicotine replacement therapy (NRT), cessation rates may be further improved, but the efficacy and safety of the combination need to be evaluated.

**Objective:**

To examine whether hospitalized smokers treated with varenicline and NRT lozenges achieve higher prolonged smoking abstinence rates compared with those treated with varenicline alone.

**Design, Setting, and Participants:**

A double-blind, placebo-controlled randomized clinical trial was conducted in adult medical or surgical inpatients of 5 Australian public hospitals with a history of smoking 10 cigarettes or more per day, interested in quitting, and available for 12-month follow-up between May 1, 2019, and May 1, 2021 (final 12-month data collection in May 2022). Data analysis was performed from June 1 to August 30, 2023.

**Interventions:**

A 12-week varenicline regimen was initiated during hospitalization at standard doses in all participants. Participants were randomized to additionally use NRT (2 mg) or placebo lozenges if there was an urge to smoke. Behavioral support (Quitline) was offered to all participants.

**Main Outcomes and Measures:**

The primary outcome was biochemically verified sustained abstinence at 6 months. Secondary outcomes included self-reported prolonged abstinence, 7-day point prevalence abstinence (3, 6, and 12 months), and medicine-related adverse events.

**Results:**

A total of 320 participants (mean [SD] age, 52.5 [12.1] years; 183 [57.2%] male) were randomized. The conduct of biochemical verification was affected by COVID-19 restrictions; consequently, the biochemically verified abstinence in the intervention vs control arms (18 [11.4%] vs 16 [10.1%]; odds ratio [OR], 1.14; 95% CI, 0.56-2.33) did not support the combination therapy. The secondary outcomes in the intervention vs control arms of 7-day point prevalence abstinence at 6 months (54 [34.2%] vs 37 [23.4%]; OR, 1.71; 95% CI, 1.04-2.80), prolonged abstinence at 12 months (47 [29.9%] vs 30 [19.1%]; OR, 1.77; 95% CI, 1.05-3.00), and 7-day point prevalence abstinence at 12-months (48 [30.6%] vs 31 [19.7%]; OR, 1.79; 95% CI, 1.07-2.99) significantly improved with the combination therapy. The self-reported 6-month prolonged abstinence (61 [38.6%] vs 47 [29.7%]; OR, 1.49; 95% CI, 0.93-2.39) favored the combination therapy but was not statistically significant. Medicine-related adverse events were similar in the 2 groups (102 [74.5%] in the intervention group vs 86 [68.3%] in the control group).

**Conclusions and Relevance:**

In this randomized clinical trial of the combination of varenicline and NRT lozenges in hospitalized adult daily smokers, the combination treatment improved self-reported abstinence compared with varenicline alone, without compromising safety, but it did not improve biochemically validated abstinence.

**Trial Registration:**

anzctr.org.au Identifier: ACTRN12618001792213

## Key Points

**Question:**

Does the combination of varenicline and nicotine lozenges improve smoking cessation rates in hospitalized smokers compared with varenicline alone?

**Findings:**

In this randomized clinical trial of 320 adult daily smokers, the combination of varenicline and nicotine lozenges initiated during hospitalization did not improve biochemically verified abstinence, the primary outcome, which was affected by COVID-19 restrictions; however, self-reported smoking abstinence at 6 and 12 months, the main secondary outcomes, were higher in the combination group than the varenicline-alone group.

**Meaning:**

Smokers wishing to quit have an additional pharmacologic treatment option in the form of varenicline and nicotine lozenges that may be safely used in combination, but additional studies may be needed to confirm its effectiveness.

## Teaser

This randomized clinical trial of adult daily smokers examines whether the combination of varenicline and a nicotine lozenge provides increased efficacy over varenicline monotherapy.

## Introduction

Varenicline is the most effective sole pharmacotherapy for smoking cessation and is as effective as combination nicotine replacement therapy (NRT) (patches plus immediate-release products).^1,2^ Varenicline was designed as a high-affinity partial agonist that mediates smoking cessation through acute, rapid interactions with α_4_β_2_ receptors that reduce the full-agonist effects of nicotine. Additionally, varenicline is trapped in neurons in α_4_β_2_ receptor–containing Golgi satellites, which results in very slow release long after nicotine is gone after smoking.^3^ However, varenicline may not completely saturate nicotinic acetylcholine receptors, producing only partial attenuation of nicotine cravings.^4^ Varenicline reduces both tonic and cue-induced cigarette cravings but does not attenuate cue-induced cravings under stress.^5^ Adding NRT to varenicline treatment may enhance receptor saturation, which may lead to decreased cigarette cravings.^4,6^ Permitting patients quitting with varenicline to use an NRT product as much as desired may enable them to better manage withdrawal symptoms and cravings, particularly in relation to stress and cue-related reinstatement of smoking.^4,5^

Three small trials^6,7,8^ have evaluated the addition of NRT patches to varenicline monotherapy, with 1 trial^7^ showing significantly improved efficacy and the other 2 trials^6,8^ showing increased, but nonsignificantly different, rates of abstinence among those receiving combination therapy. Meta-analysis^9^ of these trials demonstrated that combination therapy was associated with significantly higher abstinence compared with varenicline monotherapy in both early outcomes (4-12 weeks, 3 trials) and late outcomes (24 weeks, 2 trials). Subsequent studies^10,11^ reported mixed effects.

Immediate-release forms of NRT may be superior to patches to rapidly alleviate cravings and may be a better alternative in combination with varenicline for smoking cessation. No previous studies have evaluated the efficacy of the combination of varenicline and immediate-release NRT. Furthermore, there is a paucity of data on initiating varenicline in hospitalized smokers. Hospitalization represents a teachable moment for smoking cessation and offers a conducive environment for evaluating new treatments under medical supervision and support.^12^ This randomized clinical trial (RCT) hypothesized that a combination of varenicline and a short-term oral dose form of NRT (lozenge), provided free of cost to heavy smokers admitted to the hospital and initiated under close supervision, may offer increased efficacy over varenicline monotherapy without compromising safety.

## Methods

The VANISH (Varenicline and Nicotine Replacement Therapy for Smokers Admitted to Hospitals) RCT in hospitalized smokers compared the efficacy and safety of 2 treatments for smoking cessation: varenicline and NRT lozenges vs varenicline and placebo lozenges. This study was approved by the human research and ethics committees of all 5 participating hospitals and Monash University, and the trial was prospectively registered in the Australian New Zealand Clinical Trials Registry. All participants provided written informed consent. The study followed the Consolidated Standards of Reporting Trials (CONSORT) reporting guideline. A detailed study protocol (Supplement 1) has previously been published.^13^

### Setting and Participants

Participants in the trial were recruited from 5 smoke-free tertiary public hospitals in Australia. Inclusion criteria were adult inpatients with self-reported smoking of 10 or more cigarettes per day who were interested in quitting using pharmacotherapy and available for follow-up for 12 months between May 1, 2019, and May 1, 2021 (final 12-month data collection in May 2022). Exclusion criteria were patients already receiving NRT and varenicline, those with contraindications to NRT or varenicline (hypersensitivity to nicotine, Stevens-Johnson syndrome, or erythema multiforme), those with terminal illnesses (anticipated survival <6 months), an unstable cardiovascular condition (recent myocardial infarction or stroke within the past 3 months), or a new diagnosis of a major psychiatric illness within the past 3 months.

### Randomization and Masking

Eligible participants were randomized to 1 of the study arms using a computer-generated, stratified (by site), block (random permuted block sizes of 2 and 4) randomization list. Sealed opaque envelopes were used for the concealment of treatment allocation. Trial participants were dispensed the allocated trial medications from the pharmacy department and provided detailed counseling. Participants and the outcome assessors were masked to allocation.

### Study Treatments

Intervention group participants received varenicline (Pfizer) plus 2-mg mint-flavored NRT lozenges (Nicotinell), whereas those in the control group received varenicline plus matching placebo mint lozenges. Varenicline (both treatment arms) was initiated in the hospital using the standard recommended dosing regimen of 0.5 mg/d on days 1 to 3, 0.5 mg twice daily on days 4 to 7, and 1 mg twice daily from day 8 onward for another 11 weeks. Participants were advised to use the lozenges only if there was an urge to smoke; participants were instructed to dissolve a lozenge in their mouth every 1 to 2 hours if required but not to use more than 15 lozenges in a day. The lower strength of NRT lozenge was chosen because it was used in conjunction with varenicline. However, the dosing schedule allowed participants to take additional doses as needed. All treatments were provided free of cost to participants.

Medication information was provided to the participants on the dosing regimen, common adverse effects, emergency contact details, and process of obtaining renewed supplies. Consumer Medicines Information sheets highlighting key information on the dosing regimen and common adverse effects were provided to all participants.

At week 11, a research assistant (S.P., D.M., P.L., and J.P.) telephoned all participants, and those who self-reported continuous abstinence (defined as ≤5 cigarettes in total between weeks 2 and 11) were offered an additional 12 weeks’ free supply of varenicline. Further supply was made at the discretion of the site clinician after consideration of nicotine dependence, adherence to treatment, and any adverse effects reported by the participant.

### Quitline Support

All participants (both intervention and control) were encouraged to use behavioral support from Quitline as per Quitline standard protocols. However, using Quitline support was not mandatory for participation in the study. Each participant was offered referral by the research assistant to Quitline for additional behavioral support. Quitline made a maximum of 4 attempts to reach participants and offered the standard call-back service (maximum of 6 counseling sessions).

Automated text messages were sent to all participants by Quitline using their standard procedures, that is, once a week for the first month of treatment, then once every month. Text messages reinforced the importance of adherence to the study medicines to increase abstinence and contained emergency contact details for the participants. Participants who did not have a mobile telephone were called (with their permission) on their home telephone by the research assistant instead of sending text messages.

### Baseline and Follow-Up Interviews

Data collected from participants at baseline included demographics, medical and medication history, and tobacco use behavior. Race and ethnicity data were not captured because they were not routinely collected at participating hospitals. Follow-up interviews were conducted by a blinded research assistant, who completed telephone follow-up interviews scheduled at 3, 6, and 12 months. Participants were also contacted at weeks 1 and 3 either face to face or via telephone (if already discharged from the hospital) to evaluate the safety of and adherence to treatment. Participants’ nicotine dependence and depressive symptoms were assessed using validated scales, Heaviness of Smoking Index (HSI)^14^ and the 9-item Patient Health Questionnaire (PHQ-9),^15^ respectively.

### Primary and Secondary Efficacy Outcomes

We set the primary end point for cessation as per the Russell standard and latest Society for Research on Nicotine and Tobacco recommendations^16^: 6-month prolonged abstinence (self-report of not having smoked >5 cigarettes during the 24 weeks preceding the 6-month follow-up) with biochemical verification (expired carbon monoxide level of ≤6 ppm) using a handheld carbon monoxide breath test device (piCO Smokerlyzer; Bedfont Scientific) during a hospital or home visit. From March 2020 until December 2021, the COVID-19 pandemic significantly affected all of the study sites, and carbon monoxide breath testing was prohibited due to the potentially aerosol-generating procedure involved. However, the study continued at all sites with collection of secondary efficacy outcomes: self-reported prolonged abstinence from week 2 to 3, 6, and 12 months, 7-day point prevalence abstinence at each follow-up, self-reports of adherence to therapy, psychological distress, and medicine-related adverse events. Verified prolonged abstinence by carbon monoxide testing was done in 2022 when feasible and at longer follow-ups (6-12 months) if the participant remained abstinent. Data from 6 and 12 months were combined to give biochemically verified abstinence at the longest follow-up for each participant only once (ie, ≥6 months) and presented as the primary outcome.

### Statistical Analysis

Continuous abstinence rates at 52 weeks in previous clinical trials of varenicline were 22.5% for varenicline and 8.3% for placebo.^17^ To demonstrate an absolute difference of 15% in prolonged abstinence rate between study arms (estimate based on abstinence rates in varenicline-NRT trials)^7^ at the 5% level of significance with 80% power, 160 participants per arm were needed. A total of 320 participants were recruited from the 5 hospitals (ie, 160 individuals each in the varenicline monotherapy and varenicline plus NRT lozenge arms). The effectiveness analyses were by intention to treat, and participants lost to follow-up were regarded as smokers.^16^ Deceased participants were excluded from analyses.

Baseline characteristics of both groups were summarized and standardized differences between groups determined. Continuous variables were initially assessed for normality and expressed as means (SDs) or medians (IQRs) as appropriate. Categorical variables were summarized using numbers (percentages).

Abstinence outcomes (primary and secondary) at 3, 6, and 12 months were estimated in each treatment arm. Logistic regression models (unadjusted) were fitted to examine the efficacy of the intervention on the primary and secondary abstinence outcomes at each follow-up visit after testing homogeneity between hospitals using a random-effects meta-analysis. A 2-sided *P* < .05 was considered statistically significant.

In adjusted analysis, logistic regression models adjusted to the baseline Heaviness of Smoking Index (a clinically significant factor in the literature)^18^ were used to examine the robustness of primary analyses. With multiple imputation to account for missing data, missing values were imputed using chained equations based on sociodemographic and clinical characteristics with 30 imputed datasets used for each variable. Imputed datasets were combined after estimation using the Rubin rule.^19^ All randomized participants who had completed the 12-week course of varenicline and took at least 1 dose of the lozenges were included in the per protocol analysis.

A data safety and monitoring board provided oversight of adverse event data on a periodic basis. For the primary safety outcome, the proportions of reported adverse events occurring between treatment initiation and during the 12-month follow-up were compared between treatment groups using χ^2^ tests. Analyses were performed using SPSS software, version 23.0 (IBM Corp) or Stata software, version 17 (StataCorp LLC). Data analysis was performed from June 1 to August 30, 2023.

## Results

Participants (n = 320) had a mean (SD) age of 52.5 (12.1) years, 183 (57.2%) were male and 137 (42.8%) were female, and the mean (SD) history of smoking was 37.5 (12.9) years. Of the 2856 hospital inpatients assessed for eligibility, 1039 were suitable for inclusion, and 320 (30.8%) agreed to participate; 160 were randomized to each group (Figure). Participants’ baseline characteristics are presented in Table 1. The overall retention rates at 6 and 12 months were 77.8% (n = 249) and 70.9% (n = 227), respectively, with no imbalance in dropouts between groups. Three participants in each arm died during the study for unrelated reasons.

**Figure.**
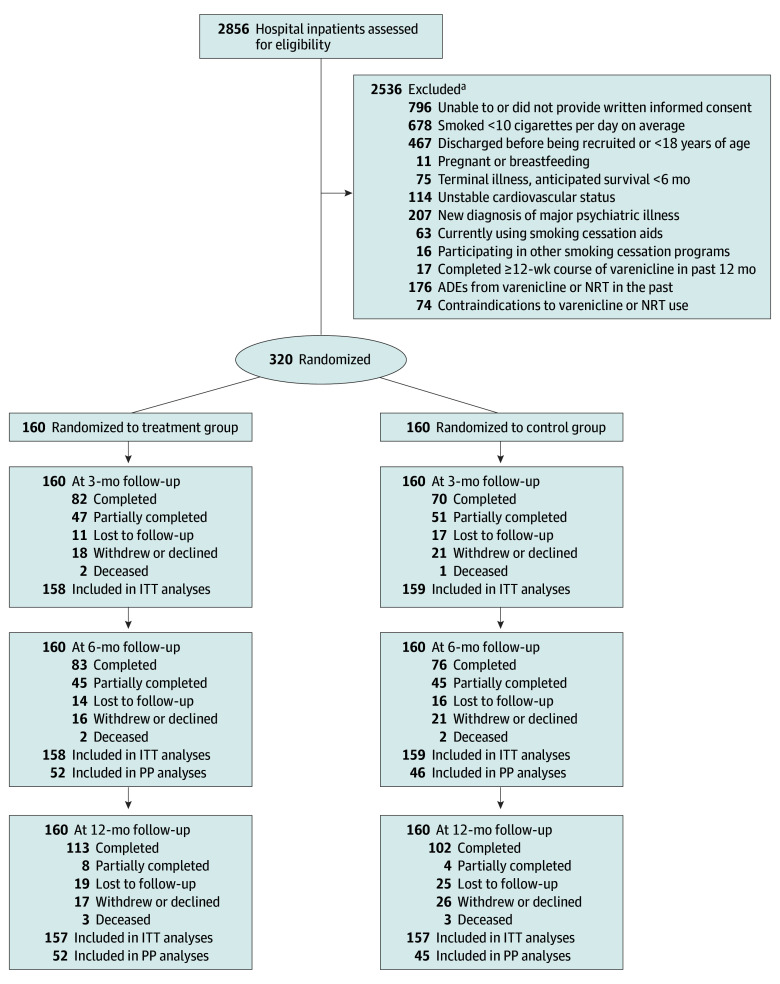
Varenicline Alone vs in Combination With Nicotine Lozenges for Smoking Cessation Among Hospitalized Smokers (VANISH) Study Flow Diagram ADE indicates adverse drug event; ITT, intention to treat; NRT, nicotine replacement therapy; PP, per protocol. ^a^More than 1 selection was possible.

**Table 1.** Baseline Characteristics of the 320 Study Participants

Characteristic	Intervention (n = 160)^a^	Control (n = 160)^a^
Age, mean (SD), y	53.4 (12.2)	51.5 (12.0)
Sex		
Male	92 (57.5)	91 (56.9)
Female	68 (42.5)	69 (43.1)
Born in Australia^b^	134 (84.3)	133 (84.2)
Mainly spoke English at home^b^	153 (96.2)	151 (95.6)
Highest educational level^b^		
No formal schooling or primary school or below	5 (3.1)	6 (3.8)
High school	94 (59.1)	91 (57.6)
Technical or further education	42 (26.4)	47 (29.7)
University education or postgraduate education	18 (11.3)	14 (8.9)
Employment status^b^		
Employed (full time, part time, or casual)	79 (49.7)	76 (48.1)
Unemployed (student, unemployed, home duties, disabled, unable to work, or retired or pensioner)	80 (50.3)	82 (51.9)
Prior hospitalization ≤6 mo previously^c^	46 (29.3)	35 (22.2)
Depression (PHQ-9)^d^		
Minimal depression (score, 1-4)	48 (32.7)	60 (40.3)
Mild depression (score, 5-9)	42 (28.6)	24 (16.1)
Moderate depression (score, 10-14)	20 (13.6)	23 (15.4)
Moderately severe depression (score, 15-19)	15 (10.2)	21 (14.1)
Severe depression (score, 20-27)	8 (5.4)	7 (4.7)
Reason for hospital admission^b^		
Cardiovascular disorder	25 (15.7)	34 (21.5)
Respiratory disorder	48 (30.2)	31 (19.6)
Digestive system disorder	29 (18.2)	25 (15.8)
Musculoskeletal disorder	15 (9.4)	19 (12.0)
Other^e^	42 (26.4)	49 (31.0)
Age at smoking onset, mean (SD), y^b^	15.8 (5.2)	16.3 (4.9)
No. of years of smoking, mean (SD)^b^	37.5 (12.9)	35.3 (12.7)
No. of cigarettes smoked per day^b^		
≤10	8 (5.0)	6 (3.8)
11-20	101 (63.5)	105 (66.5)
21-30	39 (24.5)	38 (24.1)
>30	11 (6.9)	9 (5.7)
Heaviness of Smoking Index^b^		
Low nicotine dependence (score, 0-2)	30 (18.9)	20 (12.7)
Moderate nicotine dependence (score, 3-4)	102 (64.2)	112 (70.9)
High nicotine dependence (score, 5-6)	27 (17.0)	26 (16.5)
Motivation to quit, median (IQR)^b^	8 (7-10)	8 (7-10)
Confidence to quit, median (IQR)^b^	7 (5-9)	8 (6-9)
Tried quitting at least once in the last 12 mo^b^	69 (43.4)	51 (32.3)
No. of quit attempts in the last 12 mo, median (IQR)^c^	2 (1-2)	1 (1-3)

Abbreviation: PHQ-9, 9-item Patient Health Questionnaire.

^a^ Data are presented as number (percentage) of participants unless otherwise indicated.

^b^ Data were missing for 3 participants.

^c^ Data were missing for 5 participants.

^d^ Data were missing for 24 participants.

^e^ Other reasons for hospital admission included conditions of the skin and soft tissues or reproductive, lymphatic, endocrine, urinary, or nervous systems.

### Primary and Secondary Effectiveness Outcomes

COVID-19 restrictions imposed by participating organizations on biochemical verification affected the conduct of the planned primary outcome. At 6 months, only 4 of 61 participants (6.6%) of the eligible intervention and 11 of 47 (23.4%) of the eligible control participants could complete biochemical verification. The uptake of biochemical verification at 12 months improved slightly compared with 6-month follow-up but was still low (15 of 47 [31.9%] in the intervention arm and 6 of 30 [20.0%] in the control arm).

The primary outcome of biochemically verified prolonged abstinence at 6 months or above in the intervention vs control arms (18 [11.4%] vs 16 [10.1%]; odds ratio [OR], 1.14; 95% CI, 0.56-2.33) did not reach statistical significance (*P* = .72). All secondary outcomes favored the intervention arm (combination therapy). Self-reported prolonged abstinence at 3 months (70 [44.3%] vs 52 [32.7%]; OR, 1.64; 95% CI, 1.04-2.59; *P* = .04), 7-day point prevalence abstinence at 3 months (57 [36.1%] vs 40 [25.2%]; OR, 1.68; 95% CI, 1.03-2.72; *P* = .04), 7-day point prevalence abstinence at 6 months (54 [34.2%] vs 37 [23.4%]; OR, 1.71; 95% CI, 1.04-2.80; *P* = .03), prolonged abstinence at 12 months (47 [29.9%] vs 30 [19.1%]; OR, 1.77; 95% CI, 1.05-3.00; *P* = .03), and 7-day point prevalence abstinence at 12 months (48 [30.6%] vs 31 [19.7%]; OR, 1.79; 95% CI, 1.07-2.99; *P* = .03) were all significantly higher in the intervention vs control arm, respectively. Self-reported prolonged abstinence at 6 months also favored the intervention arm, but the difference was not statistically significant (61 [38.6%] vs 47 [29.7%]; OR, 1.49; 95% CI, 0.93-2.39; *P* = .09) (Table 2).

**Table 2.** Smoking Abstinence Rates by Treatment Groups From 2 Weeks to 3, 6, and 12 Months in the Intention-to-Treat Analysis

Outcome	No. (%)			Unadjusted			Adjusted^a^	
	Intervention (n = 160)	Control (n = 160)	OR (95% CI)		*P* value	OR (95% CI)		*P* value
**Smoking abstinence at 3 mo**								
Self-reported prolonged abstinence	70 (44.3)	52 (32.7)	1.64 (1.04 to 2.59)		.04	1.67 (1.05 to 2.64)		.03
Self-reported 7-d point prevalence abstinence	57 (36.1)	40 (25.2)	1.68 (1.03 to 2.72)		.04	1.72 (1.06 to 2.80)		.03
**Smoking abstinence at 6 mo**								
Biochemically verified prolonged abstinence^b^	18 (11.4)	16 (10.1)	1.14 (0.56 to 2.33)		.72	1.24 (0.60 to 2.56)		.57
Self-reported prolonged abstinence	61 (38.6)	47 (29.7)	1.49 (0.93 to 2.39)		.09	1.52 (0.95 to 2.44)		.08
Self-reported 7-d point prevalence abstinence	54 (34.2)	37 (23.4)	1.71 (1.04 to 2.80)		.03	1.76 (1.07 to 2.89)		.03
**Smoking abstinence at 12 mo**								
Self-reported prolonged abstinence	47 (29.9)	30 (19.1)	1.77 (1.05 to 3.00)		.03	1.84 (1.08 to 3.11)		.02
Self-reported 7-d point prevalence abstinence	48 (30.6)	31 (19.7)	1.79 (1.07 to 2.99)		.03	1.85 (1.10 to 3.11)		.02

Abbreviation: OR, odds ratio.

^a^ All analyses were adjusted for baseline Heaviness of Smoking Index score.

^b^ Data from 6 to 12 months were combined to give biochemically verified abstinence at 6 months or above and presented as the primary outcome. Deceased participants at each time point were excluded from those specific analyses (3 participants at 3 months, including 2 in the intervention group and 1 in the control group; 4 participants at 6 months, including 2 in the intervention group and 2 in the control group; and 6 participants at 12 months, including 3 in the intervention group and 3 in the control group). Participants with missing outcomes were considered smokers per the Russell standard.

The 12-week course of varenicline was completed by 122 participants (38.1%), including 65 (40.6%) in the intervention arm vs 57 (35.6%) in the control arm; 257 study participants (80.3%) indicated taking at least 1 dose of varenicline, including 131 (81.9%) in the intervention arm vs 126 (78.8%) in the control arm. An additional 12-week course of varenicline was provided to 52 eligible participants (16.3%), including 25 (15.6%) in the intervention arm vs 27 (16.9%) in the control arm. A total of 180 study participants (56.3%) indicated taking at least 1 dose of NRT or placebo lozenges, including 93 (58.1%) in the intervention arm vs 87 (54.4%) in the control arm; 176 participants (55.0%) indicated taking at least 1 dose of combination study medication, including 90 (56.3%) in the intervention arm vs 86 (53.8%) in the control arm. A total of 98 participants (30.6%), including 52 (32.5%) in the intervention arm vs 46 (28.8%) in the control arm, had completed the 12-week course of varenicline and indicated taking at least 1 dose of the lozenges (NRT or placebo). The proportions of participants in each category above were not significantly different between the intervention and control groups.

A per-protocol analysis performed including those participants who had completed the 12-week course of varenicline and took at least 1 dose of the lozenges also favored the combination (eTable 1 in Supplement 2). Adjusted analyses and sensitivity analyses performed using data obtained after multiple imputation showed similar results (eTable 2 in Supplement 2).

### Exploratory Efficacy Analysis

The combination therapy significantly reduced nicotine dependence (HSI) at 6-month follow-up (median [IQR], 0 [0-2] in the intervention group vs 2 [0-3] in the control group; median difference, −2.00; 95% CI, −3.04 to −0.96). At 12-month follow-up, the combination therapy was still superior (median [IQR], 0 [0-3] in the intervention group vs 2 [0-3] in the control group; median difference, −2.00; 95% CI, −3.07 to −0.94) (Table 3). There was no difference between groups in PHQ-9 depression scores or the use of Quitline services at any visit (Table 3; eTable 3 in Supplement 2). Participants in the intervention arm self-reported greater use of nonpharmacologic interventions at both 6 and 12 months (eTable 4 in Supplement 2).

**Table 3.** HSI and PHQ-9 Score Differences From Baseline to 3, 6, and 12 Months

Outcome	Baseline			3 Months			6 Months			12 Months		
	Intervention	Control	MD (95% CI)	Intervention	Control	MD (95% CI)	Intervention	Control	MD (95% CI)	Intervention	Control	MD (95% CI)
HSI score, median (IQR)^a^	3 (3 to 4)	3.5 (3 to 4)	0 (−0.39 to 0.39)	0 (0 to 2)	0 (0 to 3)	0 (−0.64 to 0.64)	0 (0 to 2)	2 (0 to 3)	−2.0 (−3.04 to −0.96)	0 (0 to 3)	2 (0 to 3)	−2.0 (−3.07 to −0.94)
HSI score, % of participants showing improvement^b^^,^^c^	NA	NA	NA	84.0 (105/125)	80.3 (94/117)	NA	82.9 (102/123)	72.9 (86/118)	NA	78.3 (94/120)	77.7 (80/103)	NA
PHQ-9 score, median (IQR)^d^	6 (3 to 11)	5 (2 to 12)	1.0 (−0.99 to 2.99)	3.5 (0 to 11)	2 (1 to 7)	1.0 (−1.41 to 3.41)	3 (0 to 7)	2.5 (0 to 8)	0 (−2.43 to 2.43)	1 (0 to 5)	1 (0 to 5.5)	0 (−1.25 to 1.25)
PHQ-9 score, % of participants showing improvement^e^	NA	NA	NA	59.7 (43/72)	58.5 (38/65)	NA	61.3 (46/75)	65.6 (42/64)	NA	70.3 (71/101)	63.7 (58/91)	NA

Abbreviations: HSI, Heaviness of Smoking Index; MD, median difference; NA, not applicable; PHQ-9, 9-item Patient Health Questionnaire.

^a^ Data were missing for 3 participants at baseline, for 77 participants at 3 months, for 79 participants at 6 months, and for 96 participants at 12 months.

^b^ Follow-up score lower than baseline score indicates improvement.

^c^ Data were missing for 78 participants at 3 months, 79 participants at 6 months, and 97 participants at 12 months.

^d^ Data were missing for 24 participants at baseline, 172 participants at 3 months, 172 participants at 6 months, and 115 participants at 12 months.

^e^ Data were missing for 183 participants at 3 months, 181 participants at 6 months, and 128 participants at 12 months.

### Adverse Events

The most frequently reported adverse events were nausea, abnormal dreams, trouble sleeping, and headaches (Table 4). These were common during the treatment period but minor overall, with no between-group differences. The numbers of participants experiencing at least 1 adverse event during the treatment period were 102 of 137 (74.5%) in the intervention group vs 86 of 126 (68.3%) in the control group (*P* = .27) (eTable 5 in Supplement 2).

**Table 4.** Adverse Events Experienced by Treatment Group at 1 Week, 2 Weeks, 3 Months, and 6 Months

Adverse event	1 Week^a^			2 Weeks^b^			3 Months^c^			6 Months^d^			Overall^e^		
	Intervention, No. (%)	Control, No. (%)	PD (95% CI), %	Intervention, No. (%)	Control, No. (%)	PD (95% CI), %	Intervention, No. (%)	Control, No. (%)	PD (95% CI), %	Intervention, No. (%)	Control, No. (%)	PD (95% CI), %	Intervention, No. (%)	Control, No. (%)	PD (95% CI), %
Nausea	24 (19.7)	23 (20.4)	−0.7 (−10.9 to 9.6)	26 (23.4)	19 (21.8)	1.6 (−10.1 to 13.3)	15 (17.9)	13 (16.5)	1.4 (−10.2 to 13.0)	6 (8.3)	5 (6.3)	2.0 (−6.3 to 10.3)	45 (32.8)	45 (35.7)	−2.9 (−14.4 to 8.6)
Vomiting	4 (3.3)	2 (1.8)	1.5 (−2.5 to 5.5)	7 (6.3)	2 (2.3)	4.0 (−1.5 to 9.5)	3 (3.6)	2 (2.5)	1.1 (−4.2 to 6.3)	3 (4.2)	0	4.2 (−0.4 to 8.8)	12 (8.8)	6 (4.8)	4.0 (−2.0 to 10.0)
Indigestion	7 (5.7)	7 (6.2)	−0.5 (−6.5 to 5.6)	1 (0.9)	5 (5.7)	−4.8 (−10.0 to 0.4)	3 (3.6)	0	3.6 (−0.4 to 7.5)	2 (2.8)	1 (1.3)	1.5 (−3.0 to 6.0)	11 (8.0)	12 (9.5)	−1.5 (−8.3 to 5.4)
Heartburn	3 (2.5)	8 (7.1)	−4.6 (−10.1 to 0.8)	3 (2.7)	5 (5.7)	−3.0 (−8.8 to 2.7)	1 (1.2)	1 (1.3)	−0.1 (−3.5 to 3.3)	1 (1.4)	0	1.4 (−1.3 to 4.1)	6 (4.4)	11 (8.7)	−4.3 (−10.4 to 1.7)
Irritation of mouth or throat	4 (3.3)	4 (3.5)	−0.2 (−4.9 to 4.4)	5 (4.5)	4 (4.6)	−0.1 (−5.9 to 5.8)	1 (1.2)	0	1.2 (−1.1 to 3.5)	1 (1.4)	2 (2.5)	−1.1 (−5.5 to 3.3)	8 (5.8)	7 (5.6)	0.2 (−5.3 to 5.9)
Constipation	2 (1.6)	5 (4.4)	−2.8 (−7.2 to 1.6)	1 (0.9)	5 (5.7)	−4.8 (−10.0 to 0.4)	2 (2.4)	1 (1.3)	1.1 (−3.0 to 5.2)	1 (1.4)	0	1.4 (−1.3 to 4.1)	5 (3.6)	7 (5.6)	−2.0 (−7.0 to 3.2)
Weight gain	2 (1.6)	4 (3.5)	−1.9 (−6.0 to 2.2)	2 (1.8)	2 (2.3)	−0.5 (−4.5 to 3.5)	1 (1.2)	1 (1.3)	−0.1 (−3.5 to 3.3)	0	1 (1.3)	−1.3 (−3.7 to 1.2)	3 (2.2)	6 (4.8)	−2.6 (−7.0 to 1.9)
Abdominal pain	5 (4.1)	8 (7.1)	−3.0 (−8.9 to 2.9)	2 (1.8)	2 (2.3)	−0.5 (−4.5 to 3.5)	4 (4.8)	2 (2.5)	2.3 (−3.5 to 8.0)	1 (1.4)	2 (2.5)	−1.1 (−5.5 to 3.3)	11 (8.0)	11 (8.7)	−0.7 (−7.4 to 6.0)
Increased appetite	6 (4.9)	6 (5.3)	−0.4 (−6.0 to 5.2)	4 (3.6)	4 (4.6)	−1.0 (−6.6 to 4.6)	1 (1.2)	2 (2.5)	−1.3 (−5.5 to 2.8)	0	0	0	9 (6.6)	11 (8.7)	−2.1 (−8.6 to 4.3)
Headaches	8 (6.6)	7 (6.2)	0.4 (−5.9 to 6.6)	4 (3.6)	6 (6.9)	−3.3 (−9.6 to 3.1)	4 (4.8)	1 (1.3)	3.5 (−1.7 to 8.7)	1 (1.4)	1 (1.3)	0.1 (−3.5 to 3.8)	14 (10.2)	12 (9.5)	0.7 (−6.5 to 7.9)
Trouble sleeping	8 (6.6)	10 (8.8)	−2.2 (−9.1 to 4.5)	14 (12.6)	15 (17.2)	−4.6 (−14.7 to 5.4)	10 (11.9)	0	11.9 (5.0 to 18.8)	2 (2.8)	1 (1.3)	1.5 (−3.0 to 6.0)	27 (19.7)	22 (17.5)	2.2 (−7.1 to 11.6)
Abnormal dreams	16 (13.1)	15 (13.3)	−0.2 (−8.8 to 8.5)	15 (13.5)	18 (20.7)	−7.2 (−17.8 to 3.4)	10 (11.9)	9 (11.4)	0.5 (−9.3 to 10.4)	10 (13.9)	2 (2.5)	11.4 (2.7 to 20.1)	33 (24.1)	32 (25.4)	−1.3 (−11.8 to 9.1)
Mood swings	5 (4.1)	6 (5.3)	−1.2 (−6.6 to 4.2)	3 (2.7)	6 (6.9)	−4.2 (−10.3 to 1.9)	5 (6.0)	2 (2.5)	3.5 (−2.7 to 9.6)	1 (1.4)	1 (1.3)	0.1 (−3.5 to 3.8)	11 (8.0)	12 (9.5)	−1.5 (−8.3 to 5.4)
Restlessness	4 (3.3)	7 (6.2)	−2.9 (−8.4 to 2.5)	2 (1.8)	5 (5.7)	−3.9 (−9.4 to 1.5)	3 (3.6)	0	3.6 (−0.4 to 7.5)	1 (1.4)	0	1.4 (−1.3 to 4.1)	9 (6.6)	10 (7.9)	−1.3 (−7.7 to 4.9)
Dry mouth	6 (4.9)	7 (6.2)	−1.3 (−7.1 to 4.6)	4 (3.6)	5 (5.7)	−2.1 (−8.1 to 3.9)	3 (3.6)	1 (1.3)	2.3 (−2.4 to 7.0)	1 (1.4)	2 (2.5)	−1.1 (−5.5 to 3.3)	12 (8.8)	10 (7.9)	0.9 (−5.9 to 7.5)
Thoughts of self-harm	0	0	0	0	1 (1.1)	−1.1 (−3.4 to 1.1)	0	0	0	0	0	0	0	1 (0.8)	−0.8 (−2.3 to 0.8)
Changes in mood or behavior	4 (3.3)	4 (3.5)	−0.2 (−4.9 to 4.4)	4 (3.6)	7 (8.0)	−4.4 (−11.1 to 2.2)	5 (6.0)	0	6.0 (0.9 to 11.0)	0	0	0	10 (7.3)	10 (7.9)	−0.6 (−7.1 to 5.8)
Other^f^	21 (17.2)	19 (16.8)	0.4 (−9.2 to 10.0)	10 (9.0)	10 (11.5)	−2.5 (−11.0 to 6.1)	9 (10.7)	5 (6.3)	4.4 (−4.1 to 12.9)	2 (2.8)	3 (3.8)	−1.0 (−6.7 to 4.7)	37 (27.0)	30 (23.8)	3.2 (−7.3 to 13.7)

Abbreviation: PD, percentage difference.

^a^ Data were missing for 85 participants.

^b^ Data were missing for 122 participants.

^c^ Data were missing for 157 participants.

^d^ Data were missing for 169 participants.

^e^ Data were missing for 57 participants.

^f^ Other includes hiccups, dizziness, sweats, palpitations, rashes, metallic taste, and other characteristics.

## Discussion

To our knowledge, VANISH is the first reported RCT comparing the efficacy and safety of varenicline alone with the combination of varenicline and an immediate-release form of NRT in hospitalized heavy smokers. The extenuating circumstances of the COVID-19 pandemic led to small numbers of biochemical verification at 6 and 12 months, which did not yield a significant difference between study groups. However, all secondary outcomes consistently favored combination therapy. The combination also reduced nicotine dependence in participants and was well tolerated. Although this study failed to prove the superiority of the combination therapy with respect to the primary end point, it showed some potential benefits. Although the effectiveness may need to be confirmed in large clinical trials, the NRT lozenges might be considered as an ad libitum treatment for heavy smokers who continue to experience withdrawal symptoms with varenicline monotherapy.

Evaluation of combined approaches to smoking cessation with varenicline have been limited to varenicline and nicotine patches with mixed effects. Our combination has some advantages over patches (a slow, continuous nicotine delivery system), allowing use whenever needed and rapidly alleviating cravings as they are experienced. Furthermore, a previous study^10^ did not support the use of a nicotine-patch combination for an extended treatment duration. It is important to continue to evaluate the safety and efficacy of new combination therapies because no promising new molecules are currently in the pipeline.

Strenuous tobacco control policies, such as plain packaging, higher taxes, and restrictions on tobacco use in public places, have substantially reduced the smoking rates in many countries during the past few decades. The remaining smokers may represent a hard-to-change population, who may benefit from more intensive pharmacologic treatments.^20^

The adverse events reported in this trial, including nausea, abnormal dreams, trouble sleeping, and headaches, mirror those experienced in previous trials of varenicline.^10,21^ The assessment of depressive symptoms using the PHQ-9 at each follow-up visit also did not indicate any additional psychological risks. No additional risk of adverse events was identified in the combination arm, supporting the safety of combination therapy.

Commencing varenicline therapy in the hospital has been recognized as an opportunity for harnessing patient vulnerability and a teachable moment for smoking cessation.^22,23,24^ Evidence demonstrating the benefits of varenicline for smoking cessation within the hospital setting also allows the targeting of programs for higher-risk patient groups.^23,25^ Initiating the combination under a period of supervised dosing would minimize the chances of adverse effects from these medicines after discharge, promote treatment adherence, and increase the chances of achieving long-term abstinence. Targeting hospitalized heavy smokers, a challenging group in terms of engagement but likely to benefit greatly from smoking cessation, with an effective intervention could generate a large absolute number of quitters at the population level, leading to greater benefits to the community and health care systems. Higher strengths of NRT may also be considered in future studies of the combination in larger trials. Cost-effectiveness studies of the combination of varenicline and NRT in different settings would also be valuable.

### Strengths and Limitations

Our study has a few strengths and limitations. Outcome assessments were blinded, and all those who were lost to follow-up were treated as smokers. However, participants who had previously used oral NRT might have recognized the placebo lozenge given the characteristic tingling sensation from oral dosage forms of NRT. Our study was pragmatic in nature, was performed in 5 large public hospitals in challenging circumstances during a global pandemic, and used existing systems and health practitioners for the delivery of the intervention. The evidence generated from this trial in the inpatient setting for combination treatment is unique and could inform clinical practice guidelines for smoking cessation in hospitals. The smoke-free policies of Australian hospitals offered an environment conducive for abstinence, which may have assisted some of the participants to initiate varenicline (along with the lozenge) and achieve abstinence. A per-protocol analysis performed with those participants who took the 12-week course of varenicline and at least 1 dose of the lozenge showed similar trends. Participants could reduce their smoking during the first 7 days of treatment and were asked to quit completely within 2 weeks, as per the standard dosing regimen recommended for varenicline.

Adherence to varenicline is known to be poor in the general population; most (60.4%) of those who are dispensed varenicline discontinue treatment after the initiation pack, and less than 5% of those dispensed varenicline complete 12 weeks of treatment.^26^ Given the pragmatic nature of the study, the adherence rates observed in our study are on par with routine clinical practice.

The COVID-19 restrictions substantially affected biochemical verification in those self-reporting abstinence during the trial. Consequently, only a few individuals completed the carbon monoxide breath test at their 6-month follow-up, which significantly diminished the power of our preplanned primary outcome analysis. The effect of COVID-19 lockdowns and stress also might have impacted the smokers’ motivation to quit and subsequent smoking habits and quit attempts. Both varenicline and nicotine lozenges have been in the market for many years; hence, only self-reported adverse events were assessed in this study. Clinical examinations or laboratory assessments were beyond the scope of this study.

## Conclusions

In this RCT of the combination of varenicline and NRT lozenges in adult daily smokers after hospitalization, combination treatment improved self-reported abstinence compared with varenicline monotherapy, without compromising safety. The biochemically verified abstinence did not improve in this study and might be due to the effect of COVID-19 on the conduct of biochemical verifications. Additional studies to confirm its effectiveness are warranted.

## Notes

Supplement 1.
Trial Protocol

Supplement 2. **eTable 1.** Smoking Abstinence Rates by Treatment Groups From 2 Weeks to 3, 6 and 12 Months – Per Protocol Analysis **eTable 2.** Smoking Abstinence Rates From 2 Weeks to 6 Months – Sensitivity Analysis **eTable 3.** Quitline Sessions Attended by Treatment Group **eTable 4.** Self-Reported Utilisation of Other Smoking Cessation Therapies and Alternative Products **eTable 5.** Adverse Events Experienced by Treatment Group

Supplement 3.
Data Sharing Statement
