# Supplementary material for: Varenicline and Nicotine Replacement Therapy for Smokers Admitted to Hospitals: A Randomized Clinical Trial
Source: JAMA Netw Open. 2024 Jun 27;7(6):e2418120. doi: 10.1001/jamanetworkopen.2024.18120 (PMC11211956; doi:10.1001/jamanetworkopen.2024.18120)
Supplement: Supplement 1. — Trial Protocol [file jamanetwopen-e2418120-s001.pdf]

Evaluating the efficacy and safety of varenicline as a sole pharmacotherapy vs. in combination with nicotine replacement lozenges in assisting smoking cessation among hospitalised smokers: study protocol for a randomised controlled trial

Principal Investigator:

Dr. Johnson George

Monash University Australia,
Centre for Medicine Use and Safety
T +61 3 9903 9178

Document Type: Clinical Trial Protocol

Authors: Miss Rukshar Gobarani, Prof Michael J. Abramson, Prof Billie Bonevski, Dr Gregory R. Weeks, Prof Michael J. Dooley, Prof Brian J. Smith, Dr Antony Veale, Dr Ashley Webb, A/Prof Sue Kirsa, Dr Dennis Thomas, Dr Alistair Miller, Dr Rudi Gasser, Dr Eldho Paul, Ms Jacqueline Parkinson, Miss Darshana Meanger, Ms Lisa Coward, Dr Zoe Kopsaftis, Ms Olivia Rofe, Ms Paula Lee, Dr Johnson George

Release date (Version 1): **11/06/2018**

**First Revision (Version 2):**	**27/07/2018**
**Second Revision (Version 3):**	**21/08/2018**
**Third Revision (Version 4):**	**30/08/2018**
**Fourth Revision (Version 5):**	**31/08/2018**
**Fifth Revision (Version 6):**	**25/09/2018**
**Sixth Revision (Version 7):**	**02/10/2018**
**Seventh Revision (Version 8):**	**25/10/2018**
**Eight Revision (Version 9):**	**16/05/2019**
**Ninth Revision (Version 10):**	**27/06/2019**
**Tenth Revision (Version 11):**	**23/12/2019**
**Eleventh Revision (Version 12):**	**06/09/2020**

Property of Monash University Australia
Confidential
May not be used, divulged, published, or otherwise disclosed

without the consent of Monash University

Study Management Committee Members

**Principal Investigator**	**Dr. Johnson George**<br>Senior Lecturer<br>Monash University<br>Centre for Medicine Use and Safety<br>Faculty of Pharmacy and Pharmaceutical Sciences<br>PARKVILLE, VICTORIA 3052 AUSTRALIA<br>Tel: +61 399 039 178<br>E: johnson.george@monash.edu
**Coordinating Principal Investigator**	**Dr. Greg Weeks**<br>Director of Pharmacy, Pharmacy Department<br>Barwon Health<br>GEELONG, VICTORIA 3022 AUSTRALIA<br>T: +61 342 151 591<br>E: greg@barwonhealth.org.au
**Study Supervisor**	**Prof. Billie Bonevski**<br>National health and medical research concil career Development Fellow, Behavioural scientist<br>School of Medicine and Publich Health<br>Faculty of Health and Medicine<br>University of Newcastle<br>NSW 2308 AUSTRALIA<br>T: +61 (02) 4033 5710<br>E: billie.bonevski@newcastle.edu.au
**Study Supervisor**	**Prof. Michael Abramson**<br>Professor of Clinical Epidemiology<br>Department of Epidemiology and Preventative Medicine<br>School of Public Health and Preventive Medicine<br>Monash University<br>VICTORIA 3004 AUSTRALIA<br>T: +61 (3) 9903 0573<br>E: michael.abramson@monash.edu
**Study Statistician**	**Dr. Eldho Paul**<br>Department of Epidemiology and Preventive Medicine<br>School of Public Health and Preventive Medicine<br>Monash University<br>VICTORIA 3004 AUSTRALIA<br>T: +61 3 9903 0599<br>E: eldho.paul@monash.edu
**Student Researcher**	**Rukshar Gobarani**<br>T: +61 403 477 524<br>E: rukshar.gobarani@monash.edu

Study Coordinating Centre
Monash University
Centre for Medicine Use and Safety
381 Royal Parade
Parkville,
Victoria 3052
Australia

**Study Collaborators**	
**Dr. Rudi Gasser**<br>Occupational Health Physician<br>Barwon Health<br>Geelong VICTORIA<br>T: 4215 3226<br>E: rudig@barwonhealth.org.au	**Dr. Dennis Thomas**<br>Postdoctoral Research Fellow<br>Centre for Healthy Lungs<br>Hunter Medical Research Institute (HMRI)<br>The University of Newcastle (UON)<br>T: +61 2 40420199<br>E: dennis.thomas@newcastle.edu.au
**Dr. Antony Veale**<br>Senior Respiratory Consultant<br>Respiratory Medicine<br>The Queen Elizabeth Hospital<br>SOUTH AUSTRALIA 5011<br>AUSTRALIA<br>T: +61 (8) 822 260 00<br>E: antony.veale@sa.gov.au	**Dr. Ashley Webb**<br>Specialist Anaesthetist<br>Department of Anaesthesia & Acute Pain Management<br>Peninsula Health<br>VICTORIA 3199 AUSTRALIA<br>T: +61 (3) 9784 7445<br>E: awebb@phcn.vic.gov.au
**Ms Zoe Kopsaftis**<br>Research Officer<br>Respiratory Medicine and Clinical Practice Unit<br>The Queen Elizabeth Hospital<br>SOUTH AUSTRALIA 5011<br>AUSTRALIA<br>T: +61 403 041 825<br>E: zoe.kopsaftis.sa.gov.au	**Miss Darshana Meanger**<br>Pharmacist<br>Peninsula Health<br>VICTORIA 3199 AUSTRALIA<br>T: 03 9784 7602<br>E: DMeanger@phcn.vic.gov.au
**Prof. Brian Smith**<br>Respiratory Physician<br>Bendigo Health<br>BENDIGO 3550 AUSTRALIA<br>T: (03) 5454 6000<br>E: BJ.SMITH@bendigohealth.org.au	**A/Prof Sue Kirsa**<br>Director of Pharmacy<br>Monash Health<br>VICTORIA 3168 AUSTRALIA<br>T: +61 439 800 590<br>E: sue.kirsa@monashhealth.org
**Dr. Alistair Miller**<br>Head of Unit<br>General Medicine<br>Monash Medical Centre<br>VICTORIA AUSTRALIA<br>T: 9594 3066<br>E: alistair.miller@monashhealth.org	**Ms Jacqueline Parkinson**<br>Senior Pharmacist<br>Monash Health<br>VICTORIA AUSTRALIA<br>E: jacqueline.parkinson@monashhealth.org

**Mrs Olivia Rofe**<br>Program Director Pharmacy<br>VICTORIA 3128 AUSTRALIA<br>T: (03) 98953719<br>E: olivia.rofe@easternhealth.org.au	**Ms Paula Lee**<br>Senior Clinical Trials Pharmacist<br>Eastern Health<br>VICTORIA 3128 AUSTRALIA<br>T: 03 9895 3347<br>E: paula.lee@easternhealth.org.au
**Prof. Michael Dooley**<br>Director of Pharmacy<br>Alfred Health<br>Victoria 3181 AUSTRALIA<br>T: +61 (3) 9076 2408<br>E: michael.dooley@alfred.org.au	

**Data Safety and Monitoring Board (DSMB) Members**	
**Name**	**Position**
Associate Prof. Seetal Dodd	Associate Professor, Mental Health Drug and Alcohol Services University Hospital Geelong, Barwon Health
Dr. Henry Marshall	Respiratory & Sleep Medicine Physician, Holy Spirit Northside Private Hospital, QLD<br>Thoracic Physician, The Prince Charles Hospital, QLD
Associate Professor Suzie Nielsen	Deputy Director, Monash Addiction Research Centre,<br>Monash University (Peninsula Campus)

Study Sponsor

The funding for this study was derived from the grant awarded by the Global Research Awards for Nicotine Dependence (GRAND) 2017

Sources of Study medications

Varenicline (Champix®), will be an in-kind contribution from Pfizer and Nicotine replacement lozenges will be purchased from Perrigo. Placebo lozenges are similar looking mint lollies currently available in the market.

The nicotine and placebo lozenges will be repackaged and labelled by Symbion in sachets of 4 lozenges.

Contents

1. Background ... *8*

1.1 Rationale for the proposed trial ... 8

2. Aim and Objectives ... *10*

3. Study Design ... *10*

3.1 Inclusion Criteria ... 10

3.2 Exclusion Criteria ... 11

3.3 Recruitment, Initial Screening and Allocation ... 11

3.4 Initial Interview and Baseline Data ... 12

3.5 Study treatments ... 13

3.5.1 Intervention Arm ... 13

3.5.2 Comparator Arm ... 13

3.5.3 Dosing Schedules ... 13

3.6 Quitline Support and Text Messages ... 14

3.7 Concomitant Treatments ... 15

3.8 Follow-up Interviews ... 15

3.8.1 Follow-up 1: One-week from start of treatment ... 15

3.8.1 Follow-up 2: Three-weeks from start of treatment ... 16

3.8.3 Follow-up 3: Three-months from 2 weeks after treatment initiation ... 16

3.8.4 Follow-up 4: Six-months from 2 weeks after treatment initiation ... 16

3.8.5 ... 16

3.9 Assessments and Main Outcome Measures ... 16

3.9.1 Baseline Data ... 16

3.9.2 Primary Outcome Measure ... 17

3.9.3 Secondary Outcome Measures ... 17

3.10 Withdrawal Criteria ... 17

3.11 End of Trial ... 18

3.12 Reimbursement ... 18

4. Statistical Considerations ... *18*

4.1 Sample Size ... 18

4.2 Data Analysis ... 18

5. Ethics ... *19*

6. Data Safety and Monitoring Board ... *19*

Appendix A ... *21*

Appendix B ... *22*

Appendix C*... *23

1. Background

Cigarette smoking is one of the leading causes of preventable deaths worldwide (1). A report based on data from the Global Burden of Disease study indicates that in 2015, smoking killed more than 6 million people worldwide resulting in a global loss of 150 million disability adjusted life-years (2). In Australia, in the year 2016 alone, 14,900 Australians died from smoking related illnesses. That is 40 preventable deaths every day (3).

Smoking tobacco causes exposure to thousands of toxic chemicals, 70 of which are known carcinogens that have the potential to damage nearly every organ system in the human body (4). The harmful effects of these toxins are well established and include lung cancer, heart disease, chronic obstructive pulmonary disease, stroke and an exacerbation of various mental health conditions (4). Despite the myriad of healthcare risks associated with cigarette smoking, 14.7% of Australian adults aged 18 years or over smoked daily over a 12-month period in 2014-2015 (5).

An extensive body of research has established that quitting smoking has both immediate as well as long term health benefits, reducing the risk for diseases caused by smoking and improving overall wellbeing. A landmark 50-year follow-up study of 34,000 British male doctors started in 1951 provides the strongest evidence for this (6). Many participants in this cohort quit smoking subsequently thereby providing a natural experiment illustrating the impact the number of years of smoking have on health outcomes (6). Approximately two-thirds of persistent smokers in this study were estimated to have died due to their smoking (6). Among those who quit, the greatest health benefit was seen in those who quit earlier (6). Stopping smoking at the age of 50 years decreased the risk of smoking-related death by 50% and quitting at ages 60, 50, 40 or 30 years resulted in gains of about 3, 6, 9, or 10 years of life expectancy, respectively (6). Unlike complete abstinence, research shows no noticeable improvement in health outcomes and lifespan among smokers who cut down the number of cigarettes smoked each day on a long-term basis (6).

Although the daily smoking rate amongst adults 18 years or over has declined over the past decade in Australia, results of the annual Victorian surveys that monitor smoking status demonstrate a low successful quit rate due to a high rate of relapse (5, 6). These surveys monitor the attempts to quit by current smokers in the past 12 months. The survey reports that 81% of regular smokers attempted to quit in 2010, similar to the proportion reported in 2005 (6). The proportion of regular smokers who had made multiple (three or more) attempts to quit increased from 36% in 1998 to 49% in 2010 (6). Furthermore, the survey reports that approximately 77% of current smokers reported previously attempting to quit with 66% of current smokers being classified as unsuccessful quitters (6).

1.1 Rationale for the proposed trial

The development and introduction of pharmacological agents for smoking cessation has provided the much-needed assistance for those trying to quit. Various therapeutic agents are currently available to assist in achieving abstinence and a substantial body of research has demonstrated the effectiveness of such therapies in increasing abstinence rates. The most popular of these agents in Australia is nicotine replacement therapies (NRTs) available in the

form of transdermal patches, chewing gums, lozenges, inhalers, mouth sprays and sublingual tablets (6). Smoking cigarettes releases nicotine, the agent responsible for causing addiction (6). The effects of nicotine in the body are mediated via the nicotinic cholinergic receptors in the brain (7). The α4β2 receptor subtype in particular is believed to be the principal mediator of nicotine dependence. Stimulation of these ligand-gated ion channels triggers the release of various neurotransmitters in the brain which induce signals of pleasure thus reinforcing self-administration and causing dependence (7). Smoking cessation causes the emergence of withdrawal symptoms due to the reduction in the levels of nicotine in the body (7). These symptoms include but are not limited to: irritability, a depressed mood, anxiety and restlessness (7). NRTs replace some of the nicotine in the bloodstream previously derived from cigarettes but without the many thousands of other chemicals that are also present in cigarettes which are largely responsible for causing smoking-related illnesses (6).

Varenicline (Champix®), is another pharmaceutical agent indicated for use as a smoking cessation aid. It is a prescription-only medicine currently available in Australia and is subsidised via the Pharmaceutical Benefits Scheme (PBS). Varenicline has a dual mechanism of action and exerts its pharmacological effect by acting as a partial agonist at the α4β2 receptors (8). This reduces the drop in the mesolimbic dopaminergic levels that occurs during smoking cessation thereby relieving withdrawal symptoms (8). In addition to this, varenicline also antagonizes the activity of nicotine on the α4β2 receptor subtype thereby preventing the release of neurotransmitters such as dopamine in the brain and in doing so reduces feelings of pleasure from a smoking relapse (8).

To date, several studies have evaluated the efficacy and safety of NRTs and varenicline when used as monotherapy for smoking cessation. Current literature suggests that both agents are effective in assisting abstinence (9, 10). This is evident from the results of a systematic review and meta-analysis that evaluated 84 randomised controlled trials on the relative efficacies of the different pharmacological agents on abstinence at 1 year (10). The review reported that all forms of NRT and varenicline monotherapy resulted in higher smoking cessation rates at 1 year compared to controls (NRTs 70 RCTs, OR 1.71, 95% CI, 1.55-1.88, P=<0.0001, varenicline 4 RCTs, OR 2.96, 95% CI 2.12-4.12, P=<0.0001) (10).

While several studies have examined the efficacy of both agents at inducing abstinence as monotherapy, limited information is available on the efficacy and safety of combination treatment. Available evidence to date, has been limited to varenicline in combination with NRT patches, a form of long-acting NRT (9, 11, 12). Long-acting NRT delivers a constant amount of nicotine into the bloodstream over a prolonged period of time. In contrast to this, short-acting NRT (SANRT) such as gums and lozenges are administered on demand for immediate relief of cravings and withdrawal symptoms. The effects of SANRT are short-lived and repeated administration is required. The efficacy and safety of varenicline in combination with short-acting forms of NRT is yet to be known. SANRT has proven to be just as effective as NRT patches as monotherapy in assisting smokers to quit (10). Wu et al has reported a pooled OR of 1.60 for NRT gums (95% CI, 1.60, P=<0.0001) and an OR of 1.63 for NRT patches (95% CI 1.41-1.89, P=<0.0001) versus control at 1 year (10). Varenicline in combination with SANRT may, therefore, greatly enhance the rates of long-term abstinence. During the first couple of weeks of varenicline treatment patients are able to smoke, however, SANRT may allow patients to better manage their cravings and decrease the need for

smoking during this period. The proposed research project, therefore, aims to evaluate the efficacy and safety of this combination treatment.

Smoking is one of the main causes of conditions that result in hospital admissions. Hospitalisation provides an ideal environment for initiation of smoking cessation interventions. The ‘smoke-free’ environment enforced in Australian hospitals provides inpatient smokers an opportunity to attempt quitting away from their usual environmental triggers to smoke (13). During this time of increased vulnerability regarding their health, patients may be more motivated to quit and may also be more receptive to smoking cessation interventions and change in behaviour (13). This strategy may, therefore, translate to long-term abstinence that continues post-hospital discharge.

2. Aim and Objectives

This study aims to evaluate the effectiveness and safety of the combination of varenicline and NRT lozenges at assisting achievement of abstinence amongst heavy smokers admitted to six ‘smoke-free’ public hospitals in Australia.

The primary objective of the study is to compare biochemically-verified continuous abstinence in hospitalised smokers treated using varenicline plus NRT lozenge versus varenicline monotherapy.

The secondary objectives of this study are to compare:

- Self-reported 7-day point prevalence abstinence and continuous abstinence in the two treatment arms from 2 weeks to 3, 6 and 12 months after treatment initiation
- CO verified 7-day point prevalence abstinence at 6 and 12 months from treatment initiation for participants who self-report abstinence at these follow-ups
- Self-reported treatment adherence and adverse events to the study medicines at all follow-ups as well as the number of Quitline sessions attended after treatment initiation

3. Study Design

A 2-arm randomised controlled trial (RCT) with individual patients as the unit of randomisation will be carried out. Eligible inpatient smokers will be randomly allocated to either of the study groups. The trial conforms to the CONSORT guidelines for parallel group RCTs and will be registered prospectively with the ANZ clinical trials registry.

This trial will be carried out in six ‘smoke-free’ public hospitals in Australia. The study sites are Barwon Health, Peninsula Health, Eastern Health, Monash Health, Bendigo Health and The Queen Elizabeth hospital.

3.1 Inclusion Criteria

Adults aged 18 years or over, admitted to participating hospitals with a history of smoking ≥ 10 cigarettes per day on average in the four weeks prior to their hospital admission, interested in quitting smoking, willing to use pharmacotherapy and available for a 6 months follow-up post-discharge and willing/capable to provide signed informed consent will be eligible for the study. This study will not recruit any outpatient smokers. Hospitalisation

provides a great opportunity for initiating smoking cessation interventions, firstly because of the 'smoke free' policies in all study sites facilitating quitting, and secondly due to greater patient receptiveness to smoking cessation messages and interventions at a time of perceived vulnerability to negative health outcomes. Moreover, initiation of smoking cessation medications such as varenicline during hospital stay allows close monitoring of patients for any adverse events.

3.2 Exclusion Criteria

Patients who do not meet all of the above inclusion criteria, those who have a terminal illness with an anticipated survival of <6 months, those who have an unstable cardiovascular status (recent myocardial infarction or stroke within the past 3 months) or those with a new diagnosis of a major psychiatric illness (e.g. psychosis) will be excluded from the study. Patients unable to communicate in English and provide written consent will also be excluded due to the potential need to regularly communicate with the investigators during the entire trial period and the lack of funding available for interpreters. Further exclusion criteria for this study are women who are pregnant, breastfeeding or planning to become pregnant in the next 6 months, patients who are current (on the day of hospital admission) users of smoking cessation medications or approaches (i.e. NRT, varenicline, bupropion [Zyban™], clonidine, nortriptyline orelectronic nicotine cigarettes), those currently participating in other smoking cessation programs or study, patients who have completed ≥12 weeks course of varenicline in the 12 months prior to their hospitalisation, those who have had intolerable adverse reactions from the use of varenicline or NRTs in the past as well as people who have contraindications for their use (see Appendix A) (including those using medications known to have major interactions with either varenicline or NRT).

3.3 Recruitment, Initial Screening and Allocation

A trained Research Assistant (RA) will be employed at each of the six hospitals to coordinate participant recruitment. Ward staff including doctors, nurses, pharmacists and physiotherapists will be informed of the study and asked to refer all patients identified as a current smoker to the RA via the paging service. Coffee vouchers will be given to ward staff for patient referrals to the RA that result in successful recruitment. Eligible patients will also be identified by the RA through active screening of the patients' admission notes. In addition to this, flyers containing study information will be displayed in hospital wards to notify inpatients of the study. Flyers will also contain contact information of the RA at the site so that if interested, patients can discuss the study with them. Upon identification of potential study participants, the RA, in consultation with the treating medical team, will assess the patients' eligibility for the study considering their current health status and any apparent contraindications for the use of varenicline or NRTs (Appendix A). Details of this initial medical screening will be recorded by the RA. The RA will then discuss with the Nurse Unit Manager or ward nurse to assess the appropriateness in approaching the patient for an interview.

The RA will describe the project to the patient and provide a participant information sheet. When the patient has had the study explained to them and an opportunity to have any questions asked, written consent will be sought before the initial interview proceeds. Each participant will be assigned a study number and baseline data will be collected during the initial interview using the baseline data collection form. Participants will then be randomised

(1:1) using a randomisation scheme generated using a computer-generated list. Random block sizes of two and four will be used to reduce the predictability of treatment allocation. Each site will be provided with 64 envelopes containing the group allocation. The clinical trials pharmacist will open the envelopes in a sequential manner when a participant is recruited. The pharmacist will then dispense the study medicines as stated in the envelope ([varenicline and NRT lozenges] or [varenicline and placebo lozenges]) and hand these to the RA along with the envelope. The RA will then hand the medicine to the participant and provide detailed counselling. Participants will not be told whether they are receiving NRT or placebo lozenges.

3.4 Initial Interview and Baseline Data

Once informed written consent has been obtained, each participant will undergo an initial interview with a RA. A detailed data collection form/questionnaire will be used during the interview to further confirm the patients' eligibility for the study and to gather baseline data. Data gathered during the interview will include information on the participants' smoking habits, previous attempts at smoking cessation and current willingness/confidence to quit.

A caution is indicated for a number of conditions in the product information sheets of varenicline and NRT lozenges. These include diabetes mellitus, renal or hepatic impairment, gastrointestinal disease, history of seizures, psychiatric illness, pregnancy, breastfeeding and cardiovascular symptoms including myocardial infarction or stroke. A detailed medical history (current medical conditions and medications) including the presence of any contraindications or cautionary conditions will be sought during the interview. The interview will also involve an assessment of the presence of any psychological symptoms using the Patient Health Questionnaire (PHQ-9).

The NRT lozenges used in this study are sugar-free but not the placebo lozenges. Smokers with a history of diabetes will not be excluded from the study but will be counselled on the need to be vigilant and perform regular self-monitoring of blood glucose levels. The final decision to include or exclude patients with diabetes will remain with the treating medical team.

Participants with any exclusion criterion including any contraindications to varenicline or NRT lozenges will be excluded from the study and will be referred to Quitline for smoking cessation support. Those participants who are noted to have a cautionary condition (without any exclusion criterion) will be referred to an in-house clinician for further assessment. The decision on whether to include participants who have a cautionary condition is at the discretion of the treating medical team and the RA based on an evaluation of the potential risks and benefits from a patient's participation in the study. In the event of any uncertainties, the expert opinion of site coordinators will be sought.

During the baseline interview, the RA will also seek the participants' consent to contact their regular General Practitioners (GP) and Community Pharmacist (CP) to inform them of the participants' involvement in the study. This will be important to ensure the safe and appropriate delivery of healthcare services to the participant. Once written consent is obtained, the RA will contact the respective GP and CP via a telephone, email or fax.

Information regarding the participants' involvement in the project will also be noted on the hospital discharge summary.

3.5 Study treatments

There are two treatment arms in this study. All participants will be randomised to one of two treatment arms. The allocated study medications will be provided free of charge.

Brands to be used

Varenicline	Champix™
NRT lozenges	Nicaway™ 2mg lozenges
Placebo lozenges	Mint lozenges

NRT and placebo lozenges will be purchased in bulk and repackaged and labelled in sachets, each containing two lozenges.. Distribution of varenicline and repackaged lozenges will take place from Barwon Health or Symbion to the other four hospitals (study sites). All participants will be provided with 12 weeks supply of varenicline and 200 lozenges on recruitment. Renewal supplies of trial lozenges (nicotine and placebo) will be delivered to participants on their request.

It is still possible that participants with prior experience in using NRT, especially the NRT lozenges (Perrigo Inc) distinguish the active and placebo lozenges solely based on taste and texture; it will be acknowledged as one of the limitations of the study.

3.5.1 Intervention Arm

Participants in the intervention arm will receive varenicline plus NRT (lozenges) for 12 weeks. An additional 12 weeks course of varenicline will be provided (i.e. 24 weeks in total) to those who need further support (to be determined by the staff in charge of delivering the intervention based on participant's nicotine dependence, adherence to treatment, and any adverse events]. NRT lozenges/placebo will also be provided to those needing further support.

3.5.2 Comparator Arm

Participants in this arm of the study will receive varenicline plus NRT placebo (mint) lozenges for 12 weeks with an additional 12 weeks course of varenicline provided to those needing further support (i.e. 24 weeks in total).

3.5.3 Dosing Schedules

Varenicline standard dosing schedule derived from the varenicline Product Information sheet (14)

**Treatment days**	**Dosage**
1 – 3	One 0.5mg tablet once daily
4 – 7	One 0.5mg tablet twice daily

8 – onwards	One 1mg tablet twice daily

Participants will receive varenicline immediately after randomisation and will be informed to start the medication soon after they receive it. All participants will be asked to completely abstain from smoking if possible or at least reduce their smoking over the first seven days of the varenicline course and to aim to quit completely within one to two weeks of starting. Along with the medication, participants will also be given Consumer Medicines Information (CMI) sheets highlighting key information about the dosing schedule and important adverse effects to be aware of. A trained RA will give clear verbal counselling to the participants along with a lozenge instruction sheet which explains how to use the lozenges, the most common side effects, emergency contact details and information on how to obtain a renewal of their medication.

If a participant experiences severe or intolerable nausea from varenicline, a RA will check their method of administration (i.e. how they take the tablets in relation to food and water) and if needed reduce the dose as recommended by the Australian Medicines Handbook 2018 and Varenicline Product Information Sheets.

NRT and Placebo Lozenges dosing schedule

**Instructions for use of lozenges derived from Perrigo product information sheet (15)**
Lozenge (NRT or mint) should be taken only when there is an urge to smoke. Take a lozenge (2 mg) as required when you have an urge to smoke up to every 1-2 hours as needed. You must not use more than 15 lozenges in a day.<br>1. Place one lozenge on the tongue and suck until the taste becomes strong<br>2. Rest the lozenge between the gum and cheek<br>3. When the taste fades start sucking the lozenge again<br>4. Repeat this process until the lozenge completely dissolves (it takes about 30 minutes)

Participants will receive NRT or placebo lozenges immediately after randomisation (i.e. at the same time as varenicline). They will be informed to start the medication (or placebo) soon after they receive it. As with varenicline, participants will receive a Consumer Medicines Information (CMI) sheet and a lozenge instruction sheet highlighting key information about the lozenges including information on how to take the lozenges and some common side effects of which to be aware. A trained RA will give clear verbal counselling to the participants along with a lozenge instruction sheet which explains how to use the lozenges, the most common side effects, emergency contact details and information on how to obtain a renewal of their medication.

3.6 Quitline Support and Text Messages

Participants will be informed and encouraged to use behavioural support from Quitline as per Quitline standard protocols. Using Quitline support is not a requirement for participation in the study.

Participants will be sent automated text messages once a week for the first month of treatment, then once every month. Text messages will be sent by Quitline or by the RA using the ClickSend system. Text messages will reinforce the importance of adherence to the study medications, long-term abstinence and also contain emergency contact details for the participants. Participants who do not have a cell phone will be called on their land phone by the RA instead of sending text messages.

3.7 Concomitant Treatments

All participants are encouraged to inform their GPs and CPs about their participation in this study upon discharge from hospital to ensure a safe delivery of healthcare services. Throughout the study, participants will be able to take any medication as required, except for smoking cessation medications. Use of concomitant medication will be recorded at each of the study follow-ups and checked for potential interactions. The use of other smoking cessation medications including other forms of NRTs (i.e. patches or gums) is strongly discouraged during the course of the study to ensure patient safety. If a participant uses other smoking cessation medication during the study period, an appropriate record of this will be maintained. Their results will still be included in the primary and secondary analyses, however, sensitivity analysis will be performed by excluding them from the primary analysis.

3.8 Follow-up Interviews

Five follow-up interviews will be conducted over the entire course of the study: at weeks one and three of treatment initiation and at three-, six- and twelve- months from treatment initiation. The one- and three- week follow up interviews will be done by the RA involved in the participant's recruitment. These follow-ups will be conducted face-to-face for participants who are still inpatients or via telephone for participants who have been discharged. Participants who are uncontactable via telephone will be sent text messages and emails for their follow-ups. Three-, six- and twelve-month follow-up interviews will be conducted via telephone by an independent RA who is blinded to treatment allocation and who was not involved in the participant's recruitment.. A total of ten attempts will be made to contact the participant for follow-up interviews. Participants' next of kin will also be contacted if the RAs are unable to reach the participants directly. If a participant is unable to be contacted beyond this point they will be noted as 'lost to follow-up' and they will be considered as "smokers" according to the Russell Standard. Participants who successfully complete follow-ups at 3, 6 and 12 months will be reimbursed for their time via a gift voucher that will be provided at the 12-month follow-up.

3.8.1 Follow-up 1: One-week from treatment initiation

For participants who are still inpatients this interview will be done in hospital, for those who have been discharged it will be done over the telephone. The purpose of this follow-up is to check adherence to treatment medication, any adverse effects experienced and answer any participant queries. Withdrawal symptoms may be more predominant at this stage, increasing the chance of relapse. Participants may also be more inclined to use concomitant non-evidence based smoking cessation approaches such as e-cigarettes, or use additional medications such as NRT patches or gums to circumvent the withdrawal symptoms and reduce cravings. Participants will be asked about their use of such medications and details will be recorded.

3.8.1 Follow-up 2: Three-weeks from treatment initiation

For participants who are still inpatients, this interview will be done in hospital, for those who have been discharged it will be done over the telephone. The focus of this follow-up is to monitor participants' response to the study medications. Data will be collected on the side effects experienced, potentially from the use of the study medications, adherence to the study medications, the presence of any withdrawal symptoms, cravings and relapses and any changes in the participant's medical conditions or medications.

3.8.3 Follow-up 3: Three-months from treatment initiation

This follow-up will be done by a RA (blinded to treatment allocation) over the phone three months after the participant has been discharged from hospital. This follow-up will gather information on patient adherence to treatment medication, adverse effects experienced, presence of any withdrawal symptoms, cravings and relapses.

3.8.4 Follow-up 4: Six-months from treatment initiation

A RA (blinded to treatment allocation) will contact the participants at six months from hospital discharge. Participant self-reports of treatment adherence, adverse effects, withdrawal symptoms and cravings will be recorded. The number of Quitline calls made throughout the treatment period will also be recorded as well as the use of any other forms of behavioural/pharmacotherapy. Participants meeting the self-reported abstinence criteria (see 3.9.2) will be asked where they would like to take the CO breath test i.e. in hospital or by having a RA visit them at home.

3.8.5 Follow-up 5: Twelve-months **from** treatment initiation

This follow-up will be done by a RA blinded to treatment allocation. Information will be gathered on participant's smoking outcomes, cravings and withdrawal symptoms, use of the Quitline service and monitor any symptoms of depression. Participants meeting the self-reported abstinence criteria (see 3.9.2) will be asked where they would like to take the CO breath test i.e. in hospital or by having a RA visit them at home. Participants recruited prior to the extension of the trial (i.e. prior to the addition of the 12-month follow-up) will be asked to provide verbal consent for this follow-up during their six-month follow-up call. Only those participants who consent to the 12-month follow-up will receive calls at this timepoint.

3.9 Assessments and Main Outcome Measures

Data will be collected upon consent and at each of the five follow-ups.

3.9.1 Baseline Data

Information collected at the time of the baseline interview after obtaining written informed consent will include the following:

- Demographic information: contact details, country of birth, age, gender, education, employment status, living arrangement and possession of concession cards
- Smoking habit: age when they started, average number of cigarettes smoked per day, previous quitting experiences and methods used, perceptions about smoking cessation and willingness/confidence to quit

- Nicotine dependence: nicotine dependence will be assessed using the 2-item Heaviness of Smoking Index (HSI)
- Psychological distress: presence and extent of psychological distress will be assessed using the Patient Health Questionnaire (PHQ-9)
- Participant's motivation to quit smoking and confidence to quit smoking will be assessed using a 10 point scale with one being 'very low' and 10 being 'very high'
- Medical history: including a list of all current medications and medical conditions

3.9.2 Primary Outcome Measure

The primary outcome will be biochemically verified continuous abstinence from 2 weeks to 6 months from treatment initiation. A 2-week period will be allowed on treatment commencement to match the recommendation in the varenicline product information sheet. Participants who self-report abstinence (i.e. self-report smoking no more than five cigarettes, including the use of non-combustible tobacco products and electronic cigarettes) over this period (i.e. weeks 2-26) will be asked to perform a carbon monoxide (CO) breath test. CO levels will be measured by trained RAs blinded to treatment allocation, using using a hand held piCO+ Smokerlyzer (Bedfont Scientific, Maidstone, Kent, UK) during a hospital or home visit. Participants with a CO level <10ppm will be considered abstinent. Sensitivity analysis will be performed using a lower CO cut-off of <5ppm.

3.9.3 Secondary Outcome Measures

The secondary outcome measures are:

- Self-reported continuous abstinence from 2 weeks to three-, six- and twelve- months from treatment initiation
- CO verified continuous abstinence from 2 weeks to 12 months from treatment initiation for participants who self-report abstinence at this follow-up
- Self-reported 7-day point prevalence abstinence (i.e. smoking not even a puff in the past 7 days on the day of follow-up) measured from 2 weeks to 3, 6 and 12 months from treatment initiation
- CO verified 7-day point prevalence abstinence from 2 weeks to 6 and 12 months from treatment initiation for participants who self-report abstinence at these follow-ups
- Participant self-reports of withdrawal symptoms and cravings using the Mood and Physical Symptoms Scale (MPSS)
- Self-report of treatment adherence using the 8-item Tool for Adherence Behaviour Screening (TABS)
- Adverse events experienced from study medications
- Change in psychological distress
- Number of Quitline sessions attended/received
- Other health resources used including emergency department visits, GP visits (for smoking cessation), over-the-counter medicines used as well as the use of other smoking cessation therapies and alternate products

3.10 Withdrawal Criteria

All participants are strongly encouraged to complete the study, however there may be certain situations in which withdrawal from the study may be appropriate. Participants may withdraw from the study if one or more of the following occurs:

- The participant experiences any serious adverse event (SAE) or reaction due to the use of varenicline or NRT lozenges. Prior to treatment discontinuation, input from the treating medical team and end point evaluation committee will be sought in establishing the association between treatment exposure and adverse events. The DSMB will review all such cases and make the final judgement on causality.
- If a female participant becomes pregnant during the course of treatment
- If a participant's health status changes significantly and the study medications are no longer in the best interest of the participant
- The lead investigators or health professionals perceive, for any reason, that the study is no longer in the best interest of the participant

A participant wishing to withdraw from the study will be requested to fill a 'withdrawal form' for record purposes. Once withdrawn, the participant will not be contacted for further data collection, however their available data will be included in the Intension-to-treat analysis. If withdrawal is a result of an adverse drug reaction, the participant will be followed until the adverse reaction resolves or there is return to clinically acceptable medical status.

3.11 End of Trial

Upon the completion of the trial, participants will be requested to handover any remaining medications to the nearest community pharmacy for safe disposal (via The National Return and Disposal of Unwanted Medicines [RUM] program). Participants will also be guided to their community pharmacist and Quitline for support on how to stay 'smoke-free'.

3.12 Reimbursement

Participants will be compensated for travel expenses they incur for follow-up visits (e.g. travel for CO testing including parking expenses, mileage within 20km from the testing site and public transport fares). In addition to this, participants will be given a remuneration of $50 for travel to the hospital or other testing site if a CO breath test is required at the twelve-month follow-up.

4. Statistical Considerations

4.1 Sample Size

To show a difference of 15% in continuous abstinence between study arms (based on continuous abstinence rate of 23% in varenicline trials) at the 5% level of significance with 80% power, we will need 160 subjects per arm. i.e. 320 participants in total from the six hospitals, , 160 in each of the study arms. The primary analysis will be intention to treat (ITT) and lost to follow-up participants will be regarded as smokers.

4.2 Data Analysis

Data will be stored in a suitable database (e.g. REDCap™, ACCESS™) and exported for analysis in Statistical Package for Social Sciences (SPSS) (version 23.0; IBM, Armonk, NY) and Stata (ver 15, StataCorp, College Station, TX). As recommended by the Russell Standard, all randomised patients will be accounted for in the intention to treat (ITT) analysis. Participants with missing outcomes at follow-up or whose self-reported abstinence was not biochemically validated will be considered as smokers. Sensitivity analyses using multiple imputation methods will also be carried out. Deceased participants will be excluded from

analyses. In a supportive analysis of the primary efficacy endpoint, an analysis will also be conducted on the per protocol set, which excludes patients with any major protocol deviations.

Logistic regression analyses will be used to examine the efficacy of intervention on the primary outcome, after testing homogeneity between hospitals using a random effects meta-analysis. In the event of heterogeneity, a more robust model incorporating clustering by hospital will be fitted. The effect of intervention on continuous abstinence at 6 months will be tested in pre-specified subgroups (per hospital, nicotine dependence, highly motivated versus moderately motivated smokers and men versus women) using models fitted for each subgroup containing main effects for intervention and subgroup and an interaction between them. Statistical significance will be considered at a two-sided p value of 0.05.

All randomised participants who take at least one dose of the treatment medications will be included in the safety analysis. A chi-squared test will be done to compare the frequency of treatment withdrawal between the treatment and comparator arms. The number of participants discontinuing treatment prematurely for any reason will be summarised by treatment group and by reasons for discontinuation.

The incidence of all suspected adverse events will be summarised by treatment group under the following categories: type, severity, action taken and outcome. Severity of adverse events will be reported using the CTEAE grading scale (v5.0). The causality of the adverse events will be determined using the Noranjo algorithm.

5. Ethics

The trial will be conducted in compliance with the protocol, the principles of Good Clinical Practice (GCP), the National Health and Medical Research Council's (NHMRC) National Statement on Ethical Conduct in Human Research (updated 2015) and the Australian Code for the Responsible Conduct of Research (2018). Ethics committees of participating hospitals and Monash University will review and approve the study. A trained RA will approach each patient at the bedside with a plain language statement, confirm eligibility and obtain written informed consent.

Both varenicline and NRT have well established safety when used in appropriate cases. However, participant safety may still be a concern. Any potential concerns regarding eligibility will be discussed with the treating medical team. Participants in both arms will be closely monitored for any adverse effects.

6. Data Safety and Monitoring Board

A three-member independent data safety and monitoring board (DSMB) and study statistician will be set up for reviewing any adverse effects reported in the trial. It acts as an external review committee to ensure the safety of study participants and protect the scientific integrity of the trial. The DSMB regularly reviews trial safety and outcome data and makes recommendations regarding the continuation of the trial based on these data. All serious adverse events (SAEs) will be adjudicated by an end point evaluation committee, which reviews documentation related to the SAE and decides regarding its potential causal

relationship with the study drug. Suspected SAEs are also reported as required to the ethics committee of the hospital which enrolled the participant, the research ethics committee of Monash University, and to the study sponsor. In the event of any serious adverse events or if there are any safety concerns, treatment will be discontinued in consultation with the treating medical team and end point evaluation committee. Any support necessary to those affected or concerned will be provided independent of the study.

Appendix A

List of contraindications and precautions for the use of varenicline and NRT lozenges

	Contraindications	Precautions
**Varenicline**	- Hypersensitivity to the active substance or any excipients	- Irritability<br>- Depression<br>- History of psychiatric illness<br>- Predisposition to seizures (including conditions that may lower seizure threshold)<br>- History of cardiovascular disease<br>- Renal Impairment<br>- Pregnancy<br>- Breastfeeding
**NRT Lozenges**	- Non-smokers<br>- Phenylketonuria<br>- Hypersensitivity to nicotine or any other excipients<br>- Children <12 years of age	- Hemodynamically unstable patients i.e. serious arrhythmias, myocardial infarction, stroke, cerebrovascular disease<br>- Phaeochromocytoma<br>- Uncontrolled hypertension<br>- Diabetes mellitus<br>- Renal or hepatic impairment<br>- Sodium restricted diets<br>- Pregnancy<br>- Breastfeeding<br>- Gastrointestinal disease

Appendix B

List of drug interactions of varenicline and NRT lozenges:

<table>
<tr><th></th><th>Varenicline</th><th>NRT lozenges</th></tr>
<tr><td>Interactions</td><td>No clinically meaningful drug interactions have been identified

Alcohol → limited clinical information on a potential interaction between alcohol and varenicline. Increased intoxicating effects of alcohol have been reported by patients taking varenicline. Drinking alcohol may increase the risk of experiencing neuropsychiatric events during treatment with varenicline</td><td>No clinically relevant interactions between NRT and other drugs have been established but nicotine may enhance the haemodynamic effect of adenosine.</td></tr>
<tr><td colspan="3">Tobacco smoke may induce the metabolism of drugs that are catalysed by CYP 1A2 and thus when a person stops smoking this may result in slower metabolism and a subsequent rise in blood levels of these drugs. Such drugs may need dose adjustments at the smoking cessation (theophylline, imipramine, clomipramine, insulin, clozapine, olanzapine, fluvoxamine and caffeine) (15).</td></tr>
</table>

Appendix C

List of possible adverse events caused by varenicline and NRT lozenges based on severity:

<table>
<tr><th colspan="3">Varenicline adverse events</th></tr>
<tr><th>Common (rate ≥1%)</th><th>Uncommon (≥1/1,000, <1/100)</th><th>Rare but serious (≥1/10,000 to <1/1,000))</th></tr>
<tr><td>- nausea
- vomiting
- indigestion
- constipation
- abdominal pain
- increased appetite
- weight gain
- headache
- insomnia
- abnormal dreams</td><td>- mood swings
- restlessness
- muscle spasms
- dry mouth
- increased heart rate
- tremor
- increased blood pressure</td><td>- thoughts of self-harm
- changes in mood or behaviour
- rash or itchy swelling on skin
- breathing problems
- swelling of the face, lips, mouth, tongue, throat
- chest pain</td></tr>
<tr><td colspan="3">For some people stopping smoking with or without the aid of treatment has been linked to changes in behaviour, mood or thinking e.g. developing suicidal thoughts or anxiety. Some people had these symptoms when they began taking varenicline (Champix), and others developed them after several weeks of treatment or after stopping. It is not known whether these changes are related to Champix, as mood changes can also occur as a result of stopping smoking.

Drinking alcohol while taking varenicline can increase the effects of alcohol. It may also increase your risk of experiencing changes to your behaviour, thinking or mood and there have also been reports of increased feelings of being drunk while taking varenicline. Should you choose to agree to participate in this study, we recommend that you avoid drinking alcohol or be careful if drinking alcohol while taking this medicine and consult your Research Assistant or GP if you notice any changes in your behaviour, mood or thinking.</td></tr>
</table>

<table>
<tr><th colspan="2">NRT lozenges adverse events</th><th></th></tr>
<tr><th>Very Common (>1/10)</th><th>Common (>1/100; <1/10)</th><th>Uncommon (>1/1000; <1/100)</th></tr>
<tr><td>- nausea
- hiccups
- flatulence</td><td>- insomnia
- dizziness
- headache
- vomiting
- constipation
- heartburn
- indigestion
- dry mouth</td><td>- excessive thirst
- lethargy
- sore lips
- anxiety
- gastroesophageal reflux
- shortness of breath
- migraine</td></tr>
</table>

Appendix D

Study flow diagram:

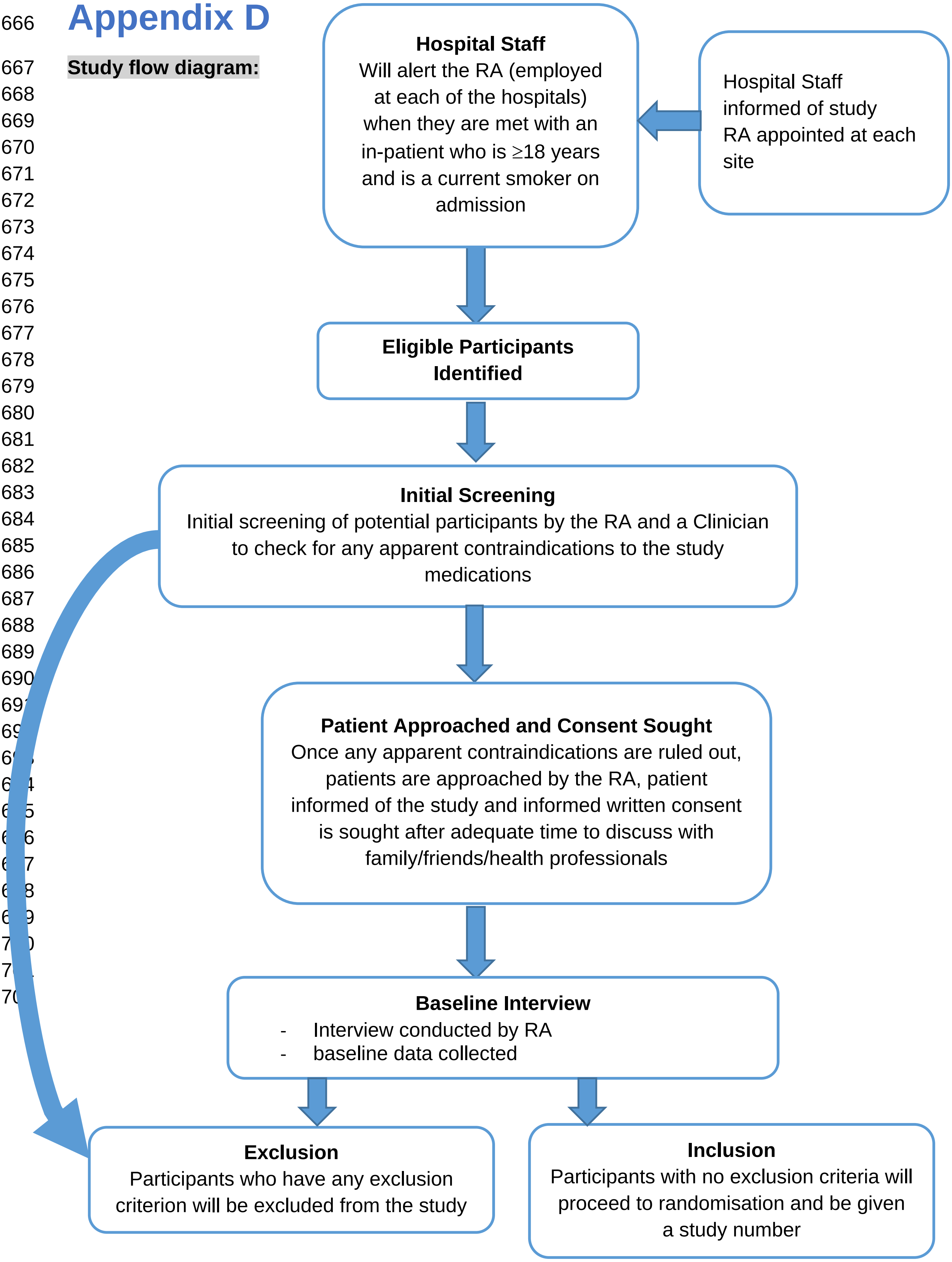

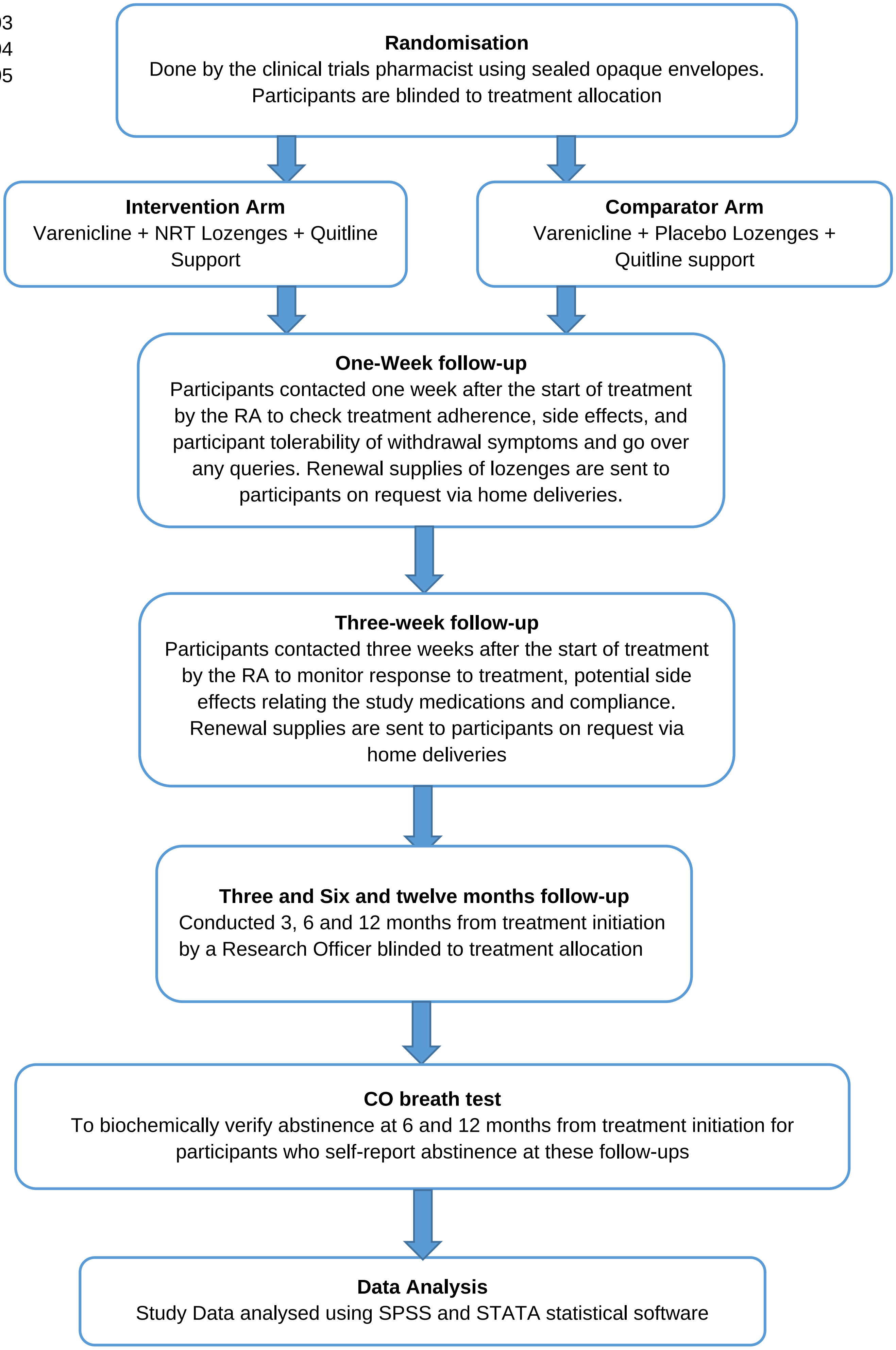
Randomisation
Done by the clinical trials pharmacist using sealed opaque envelopes.
Participants are blinded to treatment allocation
Intervention Arm
Varenicline + NRT Lozenges + Quitline Support
Comparator Arm
Varenicline + Placebo Lozenges + Quitline support
One-Week follow-up
Participants contacted one week after the start of treatment by the RA to check treatment adherence, side effects, and participant tolerability of withdrawal symptoms and go over any queries. Renewal supplies of lozenges are sent to participants on request via home deliveries.
Three-week follow-up
Participants contacted three weeks after the start of treatment by the RA to monitor response to treatment, potential side effects relating the study medications and compliance. Renewal supplies are sent to participants on request via home deliveries
Three and Six and twelve months follow-up
Conducted 3, 6 and 12 months from treatment initiation by a Research Officer blinded to treatment allocation
CO breath test
To biochemically verify abstinence at 6 and 12 months from treatment initiation for participants who self-report abstinence at these follow-ups
Data Analysis
Study Data analysed using SPSS and STATA statistical software

9. References

1. Peto R, Lopez A, Boreham J, Thun M, Heath C, Doll R. Mortality from smoking worldwide. British Medical Bulletin. 1996;52(1):12-21.
2. GBD 2015 Tobacco Collaborators. Smoking prevalence and attributable disease burden in 195 countries and territories, 1990–2015: a systematic analysis from the Global Burden of Disease Study 2015. Lancet. http://dx.doi.org/10.1016/S0140-6736(17)30819-X; 2017. (published online April 5)
3. Drope J, Schluger N, Cahn Z, Drope J, Hamill S, Islami F, Liber A, Nargis N, Stoklosa M. 2018. The Tobacco Atlas. Atlanta: American Cancer Society and Vital Strategies.
4. Drope J, Schluger N, Cahn Z, Drope J, Hamill S, Islami F, Liber A, Nargis N, Stoklosa M. 2018. The Tobacco Atlas. Atlanta: American Cancer Society and Vital Strategies.
5. Department of Health | Smoking prevalence rates [Internet]. Health.gov.au. 2018 [cited 27 July 2018]. Available from: http://www.health.gov.au/internet/publications/publishing.nsf/Content/tobacco-control-toc~smoking-rates
6. Ellerman A, Ford C, Stillman S, Quit V. [Internet]. Chapter 7 Smoking Cessation. Tobaccoinaustralia.org.au. 2012 [cited 27 July 2018].. Available from: http://www.tobaccoinaustralia.org.au/downloads/chapters/Facts_Issues--Fourth_edition/Ch7_Cessation.pdf
7. Benowitz N. Nicotine Addiction. New England Journal of Medicine. 2010;362(24):2295-2303.
8. Jiménez-Ruiz C, Berlin I, Hering T. Varenicline. Drugs. 2009;69(10):1319-1338.
9. Koegelenberg C, Noor F, Bateman E, van Zyl-Smit R, Bruning A, O'Brien J et al. Efficacy of Varenicline Combined With Nicotine Replacement Therapy vs Varenicline Alone for Smoking Cessation. JAMA. 2014;312(2):155.
10. Wu P, Wilson K, Dimoulas P, Mills E. Effectiveness of smoking cessation therapies: a systematic review and meta-analysis. BMC Public Health. 2006;6(1):1-16.
11. Hajek P, Smith K, Dhanji A, McRobbie H. Is a combination of varenicline and nicotine patch more effective in helping smokers quit than varenicline alone? A randomised controlled trial. BMC Medicine. 2013;11(1):1-7.
12. Ebbert J, Burke M, Hays J, Hurt R. Combination treatment with varenicline and nicotine replacement therapy. Nicotine & Tobacco Research. 2009;11(5):572-576.
13. Rigotti, N., Munafo, M. and Stead, L. (2007). Interventions for smoking cessation in hospitalised patients. *Cochrane Database of Systematic Reviews*. CD001837
14. Pfizer Australia Pty Ltd. Product Information. Champix (varenicline as tartrate). 2017.
15. Perrigo Australia. Herron Nicaway Lozenges (2mg and 4mg) product information sheet. 2015.

VANISH

Statistical analysis plan

Title: The efficacy and safety of varenicline alone versus in combination with nicotine lozenges for smoking cessation among hospitalised smokers (VANISH): a randomised, placebo-controlled trial

Published protocol: Gobarani RK, Abramson MJ, Bonevski B, et al. The efficacy and safety of varenicline alone versus in combination with nicotine lozenges for smoking cessation among hospitalised smokers (VANISH): study protocol for a randomised, placebo-controlled trial. BMJ open. 2020;10(10):e038184.

Investigators

- Johnson George (Chief investigator)
- Rukshar Kaizerali Gobarani (Student investigator)
- Billie Bonevski
- Michael Abramson
- Gregory Weeks
- Michael Dooley
- Dennis Thomas
- Eldho Paul (Study Statistician)
- Brian J Smith
- Antony Veale
- Ashley Webb
- Sue Kirsa
- Alistair Miller
- Rudi Gasser
- Jacqueline Parkinson
- Darshana Meanger
- Lisa Coward
- Zoe Kopsaftis
- Olivia Rofe
- Paula Lee

Aim and Objectives

This study aimed to evaluate the effectiveness and safety of the combination of varenicline and NRT lozenges at assisting achievement of abstinence amongst heavy smokers admitted to six ‘smoke-free’ public hospitals in Australia.

The primary objective of the study was to compare biochemically-verified continuous abstinence in hospitalised smokers treated using varenicline plus NRT lozenge versus varenicline monotherapy.

Sample size

Continuous abstinence rates at 52 weeks in clinical trials of varenicline were 22.5% for varenicline and 8.3% for placebo.16 To demonstrate an absolute difference of 15% in prolonged abstinence rate between study arms (estimate based on abstinence rates in varenicline-NRT trials) 6 at the 5% level of significance with 80% power, 160 subjects per arm were needed. A total of 320 participants were recruited from the five hospitals, i.e., 64 subjects from each hospital, 32 each in varenicline monotherapy and varenicline + NRT lozenges arms.

Statistical Analyses

Analyses will be performed using SPSS or Stata version 17 (StataCorp, TX, USA). Baseline characteristics of both groups will be summarised and differences between groups will be reported. Continuous variables will be initially assessed for normality and expressed as mean and standard deviation (SD) or medians and inter-quartile ranges [IQR] as appropriate. Categorical variables will be summarized using frequency counts and percentages.

Table 1: Baseline characteristics of participants (n = 320)

Variables	Intervention n = 160 (%)	Control n = 160 (%)	*P-value*
**Age in years, mean (±SD)**			
**Male**			
**Born in Australia**			
**Mainly spoke English at home**			
**Highest education**			
No formal schooling/Primary school or below			
High school			
Technical/further education			
University education/postgraduate			

**Employment status*, Unemployed**			
**Prior hospitalisation ≤6 months ago, Yes**			
**Depression (PHQ-9)**			
Minimal depression (score 1-4)			
Mild depression (score 5-9)			
Moderate depression (score 10-14)			
Moderately severe depression (score 15-19)			
Severe depression (score 20-27)			
**Reason for hospital admission**			
Cardiovascular disorder			
Respiratory disorder			
Digestive system disorder			
Musculoskeletal disorder			
Other†			
**Age at smoking onset, mean (±SD), years**			
**Number of years of smoking, median (IQR)**			
**Heaviness of smoking index**			
Low nicotine dependence (score 0-2)			
Moderate nicotine dependence (score 3-4)			
High nicotine dependence (score 5-6)			
**Motivation to quit, median [IQR]**			
**Confidence to quit, median [IQR]**			
**Tried quitting at least once in the last 12 months, n (%)**			
**Number of quit attempts in the last 12 months, median [IQR]**			

*Employed: full-time, part-time or casual; Unemployed: student, unemployed, home-duties, disabled, unable to work, retired/pensioner

†Other reasons for hospital admission include: conditions of the skin and soft tissues, reproductive, lymphatic, endocrine, urinary or nervous systems

PHQ-9: Patient Health Questionnaire-9

The effectiveness analyses will be by intention to treat (ITT) and participants lost to follow-up will be regarded as smokers. Deceased participants will be excluded from analyses.

Abstinence outcomes (primary and secondary) at 3, 6 and 12 months will be estimated in each treatment arm. Logistic regression models (unadjusted) will be used to examine the efficacy of intervention on the primary and secondary abstinence outcomes at each follow-up visit, after testing homogeneity between hospitals using a random effects meta-analysis. In the event of heterogeneity, generalised estimating equation models incorporating clustering by hospital will be fitted.

Table 2: Smoking abstinence rates by treatment groups from 2 weeks to 3, 6 and 12 months

**Outcome**	**Intervention n (%)**	**Control n (%)**	***P-value***	**Odds ratio (95% CI)**	**Adjusted odds ratio (95%CI)**
**Smoking abstinence at 3-months**					
Self-reported prolonged abstinence					
Self-reported 7-day point prevalence abstinence					
**Smoking abstinence at 6-months**					
Biochemically verified prolonged abstinence					
Self-reported prolonged abstinence					
Self-reported 7-day point prevalence abstinence					
**Smoking abstinence at 12-months**					
Biochemically verified prolonged abstinence					
Self-reported prolonged abstinence					
Self-reported 7-day point prevalence abstinence					

Deceased participants at each time-point will be excluded from those specific analyses. All analyses will be adjusted for baseline HSI score.

HSI and PHQ-9 score differences from baseline to 3, 6 and 12 months will be compared between the treatment groups using Mann-Whitney U tests.

Table 3: Heaviness of Smoking Index and Patient Health Questionnaire-9 score differences from baseline to 3, 6 and 12 months

**Outcome**	**Baseline**		**3-months**		**6-months**		**12-months**	
	Intervention	Control	Intervention	Control	Intervention	Control	Intervention	Control
**HSI score**								
**HSI score**	**----**	**----**						

**(% participants showing improvement)†**								
**PHQ-9 score**								
**PHQ-9 score (% participants showing improvement)**	**----**	**----**						

*Indicates significant differences ($p<0.05$) between the intervention and control groups. †Follow-up score lower than baseline score indicates improvement

A data safety and monitoring board (DSMB) provided oversight of adverse event data on a periodic basis. For the primary safety outcome, the proportion of reported adverse events occurring between treatment initiation and over 12-month follow-up will be compared between the treatment groups using a $\chi 2$ test or Mann-Whitney U tests, as appropriate.

Table 4: Adverse events experienced by treatment group

**Outcome**	**Intervention**	**Control**	***P-value***
Adverse events experienced at week 1*, n (%)<br>Adverse events experienced at week 1*, Median and IQR			
Adverse events experienced at week 2†, n (%)<br>Adverse events experienced at week 2†, Median and IQR			
Adverse events experienced at 3-months‡, n (%)<br>Adverse events experienced at 3-months‡, Median and IQR			

Adverse events experienced at 6-months[§], n (%)<br>Adverse events experienced at 6-months[§], Median and IQR			
Adverse events experienced from week 2 to 6-months [#], n (%)			

Table 5: Adverse events experienced by treatment group at 1-week, 2-week, 3-months and 6-months

**Adverse events**	**1-week**[*]		**2-week**[†]		**3-months**[‡]		**6-months**[§]		**Overall**[#]	
	Intervention	Control	Intervention	Control	Intervention	Control	Intervention	Control	Intervention	Control
Nausea										
Vomiting										
Indigestion										
Heartburn										
Irritation of the mouth or throat										
Constipation										
Weight gain										
Abdominal pain										
Increased appetite										
Headaches										
Trouble sleeping at night										
Abnormal dreams										
Mood swings										
Restlessness										
Dry mouth										
Thoughts of self-harm										
Changes in mood or behaviour										
Other**										

**Other includes: hiccups; dizziness; sweats; palpitations; rashes; metallic taste; etc.

Per protocol analysis: All randomized participants who took at least one dose of the treatment medications will be included in the per protocol analysis.

Table 6: Smoking abstinence rates by treatment groups from 2 weeks to 3, 6 and 12 months – Per protocol analysis

**Outcome**	**Intervention N = n (%)**	**Control N = n (%)**	**Odds ratio (95% CI)**
**Smoking abstinence at 3-months**			
Self-reported prolonged abstinence			
Self-reported 7-day point prevalence abstinence			
**Smoking abstinence at 6-months**			
Biochemically verified prolonged abstinence			
Self-reported prolonged abstinence			
Self-reported 7-day point prevalence abstinence			
**Smoking abstinence at 12-months**			
Biochemically verified prolonged abstinence			
Self-reported prolonged abstinence			
Self-reported 7-day point prevalence abstinence			

Note: Deceased participants at each time-point will be excluded from those specific analyses.

Quitline sessions attended will be compared between the treatment groups using a χ2 test.

Table 7: Quitline sessions attended by treatment group

**Outcome**	**Intervention**	**Control**	***P-value***
Quitline session/s attended till Week 1*, n (%)			
Quitline session/s attended till 3-months[†], n (%)			
Quitline session/s attended till 6-months[‡], n (%)			
Quitline session/s attended till 12-months[§], n (%)			

Utilisation of other smoking cessation therapies and alternative products will be compared between the treatment groups using a χ2 test.

Table 8: Self-reported utilisation of other smoking cessation therapies and alternative products

**Treatments**	**Intervention**	**Control**	***P-value***
**3-months follow-up**			
Nicotine replacement therapy			
Bupropion			
Non-pharmacological assistance[*]			
**6-months follow-up**			
Nicotine replacement therapy			
Bupropion			
Non-pharmacological assistance[*]			
**12-months follow-up**			
Nicotine replacement therapy			
Bupropion			
Non-pharmacological assistance[*]			

*Non-pharmacological assistance includes acupuncture, hypnotherapy, quit smoking group, etc.
