# Supplementary material for: Varenicline and Nicotine Replacement Therapy for Smokers Admitted to Hospitals: A Randomized Clinical Trial
Source: JAMA Netw Open. 2024 Jun 27;7(6):e2418120. doi: 10.1001/jamanetworkopen.2024.18120 (PMC11211956; doi:10.1001/jamanetworkopen.2024.18120)
Supplement: Supplement 2. — eTable 1. Smoking Abstinence Rates by Treatment Groups From 2 Weeks to 3, 6 and 12 Months – Per Protocol Analysis eTable 2. Smoking Abstinence Rates From 2 Weeks to 6 Months – Sensitivity Analysis eTable 3. Quitline Sessions Attended by Treatment Group eTable 4. Self-Reported Utilisation of Other Smoking Cessation Therapies and Alternative Products eTable 5. Adverse Events Experienced by Treatment Group [file jamanetwopen-e2418120-s002.pdf]

Supplementary Online Content

Weeks GR, Gobarani RK, Abramson MJ, et al. Varenicline and Nicotine Replacement Therapy for Smokers Admitted to Hospitals (VANISH): a randomized clinical trial. *JAMA Netw Open*. 2024;7(6):e2418120. doi:10.1001/jamanetworkopen.2024.18120

eTable 1. Smoking Abstinence Rates by Treatment Groups From 2 Weeks to 3, 6 and 12 Months – Per Protocol Analysis

eTable 2. Smoking Abstinence Rates From 2 Weeks to 6 Months – Sensitivity Analysis

eTable 3. Quitline Sessions Attended by Treatment Group

eTable 4. Self-Reported Utilisation of Other Smoking Cessation Therapies and Alternative Products

eTable 5. Adverse Events Experienced by Treatment Group

This supplementary material has been provided by the authors to give readers additional information about their work.

© 2024 Weeks GR et al. *JAMA Network Open.*

eTable 1: Smoking abstinence rates by treatment groups from 2 weeks to 3, 6 and 12 months – Per protocol analysis

Outcome	Intervention N = 52 n (%)	Control N = 46 n (%)	Odds ratio (95% CI)
**Smoking abstinence at 3-months**			
Self-reported prolonged abstinence	30 (57.7)	26 (56.5)	1.01 (0.45 to 2.27)
Self-reported 7-day point prevalence abstinence	25 (48.1)	14 (30.4)	2.03 (0.88 to 4.69)
**Smoking abstinence at 6-months**			
Biochemically verified prolonged abstinence	6 (11.5)	4 (8.7)	1.49 (0.39 to 5.75)
Self-reported prolonged abstinence	28 (53.8)	21 (45.7)	1.35 (0.61 to 3.01)
Self-reported 7-day point prevalence abstinence	22 (42.3)	14 (30.4)	1.65 (0.71 to 3.81)
**Smoking abstinence at 12-months**			
Self-reported prolonged abstinence	21 (40.4)	10 (22.2)	2.28 (0.92 to 5.61)
Self-reported 7-day point prevalence abstinence	22 (42.3)	11 (24.4)	2.22 (0.92 to 5.34)

Note: Deceased participants at each time-point were excluded from those specific analyses. All analyses were adjusted for baseline heaviness of smoking index (HSI) score.

© 2024 Weeks GR et al. *JAMA Network Open.*

eTable 2: Smoking abstinence rates from 2 weeks to 6/12 months – Sensitivity analysis

Outcome	Multiple imputation**	
	Odds ratio (95% CI)	*P-value*
**Smoking abstinence at 6/12-months**		
Biochemically verified prolonged abstinence	1.16 (0.57−2.36)	0.69

Note: Deceased participants at each time-point were excluded from those specific analyses (four participants at 6-months and six participants at 12-months).

**Missing outcomes were imputed using multiple imputation with age, gender, highest education, baseline heaviness of smoking index, baseline confidence and motivation to quit smoking as imputation variables

eTable 3: Quitline sessions attended by treatment group

Outcome	Intervention	Control	*P-value*
Quitline session/s attended till Week 1*, n (%)	43 (34.1)	38 (33.0)	0.859
Quitline session/s attended till 3-months†, n (%)	57 (53.8)	50 (49.0)	0.493
Quitline session/s attended till 6-months‡, n (%)	60 (56.6)	52 (52.0)	0.507
Quitline session/s attended till 12-months§, n (%)	63 (50.8)	53 (47.7)	0.640

*Missing data n = 79; †Missing data n = 112; ‡Missing data n = 114; §Missing data n = 85

© 2024 Weeks GR et al. *JAMA Network Open.*

eTable 4: Self-reported utilisation of other smoking cessation therapies and alternative products

Treatments	Intervention	Control	*P-value*
**3-months follow-up**			
Nicotine replacement therapy	7 (4.4)	5 (3.1)	0.556
Bupropion	0 (0.0)	1 (0.6)	0.317
Non-pharmacological assistance*	14 (8.8)	8 (5.0)	0.185
**6-months follow-up**			
Nicotine replacement therapy	9 (5.6)	11 (6.9)	0.644
Bupropion	0 (0.0)	1 (0.6)	0.317
Non-pharmacological assistance*	20 (12.5)	9 (5.6)	**0.032**
**12-months follow-up**			
Nicotine replacement therapy	16 (10.0)	15 (9.4)	0.850
Bupropion	0 (0.0)	1 (0.6)	0.317
Non-pharmacological assistance*	28 (17.5)	13 (8.1)	**0.012**

*Non-pharmacological assistance included acupuncture, hypnotherapy, quit smoking group, etc.

© 2024 Weeks GR et al. *JAMA Network Open.*

eTable 5: Adverse events experienced by treatment group

Outcome	Intervention	Control	*P-value*
Adverse events experienced at week 1*, n (%)	67 (54.9)	59 (52.2)	0.678
Adverse events experienced at week 1*, Median and IQR	1 [0.0 – 1]	1 [0.0 – 1]	0.929
Adverse events experienced at week 2†, n (%)	60 (54.1)	51 (58.6)	0.520
Adverse events experienced at week 2†, Median and IQR	1 [0.0 – 1]	1 [0.0 – 2]	0.382
Adverse events experienced at 3-months‡, n (%)	35 (41.7)	28 (35.4)	0.415
Adverse events experienced at 3-months‡, Median and IQR	0 [0.0 – 1]	0 [0.0 – 1]	0.198
Adverse events experienced at 6-months§, n (%)	15 (20.8)	13 (16.5)	0.489
Adverse events experienced at 6-months§, Median and IQR	0 [0.0 – 0.0]	0 [0.0 – 0.0]	0.458
Adverse events experienced from week 2 to 6-months #, n (%)	102 (74.5)	86 (68.3)	0.266

*Missing data n = 85; † Missing data n = 122; ‡Missing data n = 157; §Missing data n = 169; # Missing data n = 57

© 2024 Weeks GR et al. *JAMA Network Open.*
