# Supplementary material for: Varenicline and Nicotine Replacement Therapy for Smokers Admitted to Hospitals: A Randomized Clinical Trial
Source: JAMA Netw Open. 2024 Jun 27;7(6):e2418120. doi: 10.1001/jamanetworkopen.2024.18120 (PMC11211956; doi:10.1001/jamanetworkopen.2024.18120)
Supplement: Supplement 3. — Data Sharing Statement [file jamanetwopen-e2418120-s003.pdf]

Data Sharing Statement

Weeks. Varenicline and Nicotine Replacement Therapy for Smokers Admitted to Hospitals. *JAMA Netw Open*. Published June 27, 2024. doi:10.1001/jamanetworkopen.2024.18120

Data
Data available: Yes
Data types: Deidentified participant data
How to access data: johnson.george@monash.edu
When available: With publication

Supporting Documents
Document types: None

Additional Information
Who can access the data: researchers whose proposed use of the data has been approved
Types of analyses: For research purpose only
Mechanisms of data availability: after approval of a proposal
Any additional restrictions: None
